# Locus coeruleus co‐activation patterns at rest show higher state persistence in patients with dissociative seizures: A Pilot Study

**DOI:** 10.1002/epi4.13050

**Published:** 2024-10-07

**Authors:** Samantha Weber, Johannes Jungilligens, Selma Aybek, Stoyan Popkirov

**Affiliations:** ^1^ Department of Neurology, Psychosomatic Medicine Unit Inselspital Bern University Hospital, University of Bern Bern Switzerland; ^2^ Department of Psychiatry, Psychotherapy and Psychosomatics University of Zurich, Psychiatric University Hospital Zurich Zurich Switzerland; ^3^ Translational Imaging Center (TIC) Swiss Institute for Translational and Entrepreneurial Medicine Bern Switzerland; ^4^ Department of Neurology University Hospital Knappschaftskrankenhaus, Ruhr University Bochum Bochum Germany; ^5^ Faculty of Science and Medicine University of Fribourg Fribourg Switzerland; ^6^ Department of Neurology University Hospital Essen Essen Germany

**Keywords:** Co‐activation pattern, conversion disorder, coupling, dynamic functional connectivity, functional/dissociative seizures, locus coeruleus, PNES, psychogenic nonepileptic seizures

## Abstract

**Objective:**

Dissociative seizures are paroxysmal disruptions of awareness and behavioral control in the context of affective arousal. Alterations in stress‐related endocrine function have been demonstrated, but the timescale of dissociation suggests that the central locus coeruleus (LC) noradrenergic system is likely pivotal. Here, we investigate whether LC activation at rest is associated with altered brain network dynamics.

**Methods:**

A preliminary co‐activation pattern (CAP) analysis of resting‐state functional magnetic resonance imaging (fMRI) in 14 patients with dissociative seizures and 14 healthy controls was performed by using the LC as a seeding region. The red nucleus served as a control condition. Entry rates, durations, and state transition probabilities of identified CAPs were calculated. Analyses were corrected for demographic, technical, and clinical confounders including depression and anxiety.

**Results:**

Three LC‐related CAPs were identified, with the dominant two showing inverse activations and deactivations of the default mode network and the attention networks, respectively. Analysis of transition probabilities between and within the three CAPs revealed higher state persistence in patients compared to healthy controls for both CAP2_LC_ (Cohen's *d* = −0.55; *p* = 0.01) and CAP3_LC_ (Cohen's *d* = −0.57; *p* = 0.01). The control analysis using the red nucleus as a seed yielded similar CAPs, but no significant between‐group differences in transition probabilities.

**Significance:**

Higher state persistence of LC‐CAPs in patients with dissociative seizures generates the novel hypothesis that arousal‐related impairments of network switching might be a candidate neural mechanism of dissociation.

**Plain Language Summary:**

Dissociative seizures often arise during high affective arousal. The locus coeruleus is a brain structure involved in managing such acute arousal states. We investigated whether the activity of the locus coeruleus correlates with activity in other regions of the brain (which we refer to as “brain states”), and whether those brain states were different between patients with dissociative seizures and healthy controls. We found that patients tended to stay in certain locus coeruleus‐dependent brain states instead of switching between them. This might be related to the loss of awareness and disruptions of brain functions (“dissociation”) that patients experience during seizures.

**Key points**

- Dissociative seizures often occur immediately after a stressor, suggesting fast‐acting arousal‐mechanisms such as locus coeruleus activation.
- Patterns of brain‐wide co‐activation with the locus coeruleus show three distinct patterns.
- Patients with dissociative seizures showed tendencies to remain in certain co‐activation patterns longer than healthy controls.
- This pilot study generates novel hypotheses about locus coeruleus mediated impairments in network switching in dissociative seizures.

## 1 INTRODUCTION

Dissociative seizures, also called functional or psychogenic nonepileptic seizures, are paroxysms of altered awareness and impaired behavioral control that often arise in the context of affective arousal.^1^ Characteristic involuntary movement patterns ranging from immobility to convulsions are both a source of misdiagnosis (as epileptic or syncopal attacks), and the basis for correct semiological diagnosis by experts.^2^ Less is known about the subjective phenomenology during dissociative seizures, as awareness and recollection can be intrinsically impaired.^3^, ^4^ Patients report autonomic symptoms of arousal^5^ as well as experiences that reflect temporary disruptions of integrative higher‐order functions such as bodily awareness, behavioral control, and sense of agency.^6^ Often subsumed under the psychopathological category of “dissociation,” this multifaceted disintegration of mental functioning has remained underexplored from a neurophysiological perspective.

In general, the dynamic implementation of higher‐order brain function is best understood through the lens of large‐scale network activity. The complex interplay between self‐referential and goal‐directed processing, guiding awareness and attention between externally and internally generated representations and allowing for quick, responsive, and self‐aware behavior, is reflected in adaptive switching between intrinsic brain networks such as the default mode network, the executive control network, the dorsal and ventral attentional networks, and others.^7^ While neuroimaging studies in patients with dissociative seizures identified a range of structural alterations without a specific emerging pattern, alterations in network connectivity have repeatedly been reported for the default mode and the salience network in the overarching group of functional neurological disorder^8^, ^9^ as well as in some studies in patients with dissociative seizures.^10^, ^11^, ^12^ In a previous study on a larger cohort of patients with mixed functional neurological disorder, we found co‐activation patterns of the insula with the salience, the somatomotor, and executive control network, and co‐deactivations with the default mode network.^13^ It has also been proposed that functional neurological disorder is characterized by alterations in network dynamics, that is, the interplay between those overarching networks.^8^

One way in which acute arousal can regulate such short‐term network dynamics is through central noradrenergic activation via the locus coeruleus (LC), a small brainstem nucleus that is the sole source of synaptic noradrenaline in the central nervous system and projects widely into the neocortex.^14^ Besides the slower‐acting hypothalamic–pituitary–adrenal axis (with cortisol as its end‐product), the LC is at the core of a much faster‐acting stress system. Phasic LC burst activity facilitates attention toward external saliency and is best enabled within an intermediate level of tonic baseline activity during arousal.^14^ Both lower and higher rates of tonic activity (associated with hypo‐ and hyper‐arousal) favor self‐referential mentation and disengagement from the environment.^15^, ^16^, ^17^

Arousal reactions on medium and large timescales have been extensively studied in patients with dissociative seizures, for example, by investigating cortisol levels at baseline or after stressors, or by investigating associated neural activation patterns.^18^, ^19^, ^20^, ^21^, ^22^, ^23^, ^24^ Importantly, results from neuroendocrine analyses have so far yielded inconsistent results. Dissociative seizures can occur after prolonged periods of stress or rumination, but they are often triggered acutely in situations of escalating arousal. This suggests that a fast‐acting mechanism is involved rather than the medium‐latency cortisol response, which peaks around 20 minutes after arousal. It thus seems reasonable to assume a faster‐acting stress system such as the central noradrenergic LC‐system to have a pivotal role in the modulation of brain state micro‐dynamics that underly dissociation and dissociative seizures. This idea has not yet been explored in dissociative seizures or functional neurological disorder more broadly, but research on post‐traumatic stress disorder and panic disorder, two conditions which are often comorbid with dissociative seizures, shows that LC hyperreactivity and dysregulation of the noradrenergic network are important pathophysiological factors.^25^, ^26^

Designed as an initial pilot study to assess how LC activation affects global network activity we used co‐activation pattern (CAP) analyses,^13^, ^27^, ^28^ a neuroimaging technique that examines patterns of brain co‐activation that reappear throughout a resting‐state scan in relation to the activation of a specified seed region. These spatial co‐activation maps are clustered into a small number of discrete CAPs, to which each map is then assigned. The temporal dynamics of each individual CAP within a resting‐state period can then be calculated, reflecting global brain dynamics and their alterations. To explore the role of LC activation in brain network activity, we investigate the entry rates, durations, and, most importantly, transition probabilities of LC‐CAPs of patients with dissociative seizures in comparison with healthy controls. To support the specificity of our findings, we adjust for demographic, technical, and clinical factors and conduct a control analysis with a different seed region, the red nucleus.

## 2 MATERIALS AND METHODS

### 2.1 Participants

Aiming at investigating the role of the LC in patients with dissociative seizures, this exploratory preliminary analysis selected those patients from a previously published study of mixed functional neurological disorder^19^ who presented with dissociative seizures (*N* = 14). In addition, 14 sex‐matched healthy controls of similar age (matched at 5‐year margin) were selected. Table 1 summarizes key characteristics, and further details regarding the study design are published elsewhere.^19^ All patients had a diagnosis of dissociative seizures and no neurological comorbidity (seven probable, three clinically established, and four documented, according to diagnostic criteria of LaFrance^29^). To compare the two groups and to serve as covariates of no‐interest in our analyses, we collected (1) concomitant psychotropic medication (i.e., benzodiazepines, opioids, antidepressants, neuroleptics, and anti‐seizure medication), which was dichotomized (intake yes/no), (2) anxiety using the State–Trait Anxiety Inventory (STAI^30^), and (3) depression using the Beck's Depression Inventory II (BDI^31^). Number of seizures within the past 4 weeks were collected for each patient. The Bern Ethics Committee approved the study (SNCTP000002289), which was conducted according to the Declaration of Helsinki, and written informed consent was collected from all subjects.

**TABLE 1:** Demographic, clinical, and imaging data of patients with dissociative seizures and healthy controls.

	Dissociative seizure patients (*N* = 14)	Healthy controls (*N* = 14)	Statistics
Demographic data			
Age, mean (SD), years, [range]	34.3 (11.6), [20–58]	34.7 (11.6), [23–54]	*Z* = −0.09, *p* = 0.93
Sex (females/males)	11/3	11/3	NA
Clinical data			
Duration of illness (in months, SD)	35.0 (24.8)	NA	
Number of seizures (mean last 4 weeks, SD)	6.56 (16.41)	NA	
Additional functional neurological symptoms	7 Weakness 3 Gait disorder 1 Tremor 3 Speech disorder	NA	
Psychotropic medication (yes/no)	6/8	0/14	NA
BDI score, mean (SD)	12.2 (9.92)	2.93 (4.78)	***Z* = −3.51, *p* = 0.0004**
STAI‐S score, mean (SD)	38.1 (10.6)	31.6 (6.65)	*t*(21.87) = 1.94, *p* = 0.06
Technical data			
Number of excluded Frames, mean (SD)^a^	29.2 (45.6)	4.5 (10.2)	***Z* = −3.82, *p* = 0.0001**
Number of selected frames for CAPs analysis, mean (SD)	61.3 (6.42)	57.3 (11.2)	*Z* = −1.21, *p* = 0.22

Abbreviations: BDI, Beck's Depression Inventory; STAI‐S, State–Trait Anxiety Inventory (State); SD, standard deviation; ns, not significant; NA, not applicable.

^a^ Based on Power's framewise displacement criterion.

Bold indicates significance values (*p*) are provided in the third column.

### 2.2 Neuroimaging acquisition and pre‐processing

A 3‐Tesla scanner (Magnetom Prisma, Siemens, Germany) was used to record resting‐state functional and structural MRI data. For anatomical imaging, a sagittal‐oriented T1‐weighted 3D‐MPRAGE sequence (TR = 2330 ms, TE = 3.03 ms, TI = 1100 ms, matrix 256 × 256, FOV 256 × 256 mm, flip angle 8°, resolution 1 mm^3^ isotropic, TA = 5:27 min) was acquired for all subjects.^32^ Functional imaging data were acquired using a whole‐brain interleaved multi‐slice BOLD echo‐planar‐imaging (EPI) sequence (TR = 1300 ms; TE = 37 ms, flip angle = 52°, FOV = 230 mm, voxel size = 2.2 mm^3^ isotropic, TA = 6:39 min, for a total of 300 functional volumes). During the resting‐state acquisition, participants were fixating a white cross on a black background. To reduce head motion, participants' heads were stabilized using foam cushions. None of the participants reported on scanner‐related issues (e.g., drowsiness and dizziness) after leaving the scanner. Imaging data were pre‐processed using SPM12 (https://www.fil.ion.ucl.ac.uk/spm/software/spm12/) in MATLAB (R2017b, MathWork Inc., Natick, USA). Functional volumes were realigned and co‐registered to the structural T1 volume. They were detrended and covariates of no‐interest were regressed out, which included constant, linear, and quadratic trends, average white matter/cerebrospinal fluid time courses, motion artifacts, and global signal. Despite of global signal regression might be controversial, we applied it to the pre‐processing pipeline as it greatly improved the quality of the data. When not including global signal regression, the data were affected by too much noise particularly in tissue boundary regions. Functional data were filtered using a high‐pass filter at 0.01 Hz. Lastly, functional volumes were warped into MNI standard space and smoothed using a spatial Gaussian kernel of 5 mm full width at half maximum. Resting‐state fMRI data were inspected for exceeding translation and rotation in the *x*‐, *y*‐, and *z*‐directions. For the subsequent analysis, individual frames were scrubbed using Power's framewise displacement (FD) criterion^33^ at a threshold of FD > 0.5 mm and *z*‐scored. The analyses were restricted to gray‐matter voxels only.

### 2.3 Functional network dynamics

We analyzed spatial and temporal characteristics of the dynamic LC network derived from healthy controls using a seed‐based CAP analysis according to Bolton et al^34^ using the 7T probabilistic atlas of the human LC of Ye et al.^35^ CAP analysis was performed as follows: (1) Using the data from all healthy controls as reference population (which is advised when activity patterns are assumed to differ strongly from those of a patient population^34^, ^36^), we selected in each participant only those functional volumes in which the LC was highly active, that is, corresponding to high‐amplitude events at a threshold of 0.84 SD (80th percentile). (2) The optimal number of clusters *K* (ranging from *K* = 2:15) was identified using a consensus clustering approach on the remaining functional volumes, for which the stability of the individual cluster sizes were evaluated using the proportion of ambiguously clustered pairs (PAC) and by visually inspecting the individual consensus matrices (see Supplementary Material). At which a higher value for 1−PAC as well as crisper boundaries between the individual clusters reflected in the consensus matrices reflected a higher stability. Thus, *K* = 3 was regarded as most stable cluster size. (3) Final clustering into three CAPs was performed based on the 1−PAC and consensus matrices using *k*‐means clustering. (4) Each individual frame of the patients was assigned to its most similar CAP (derived from healthy controls) through a matching process. This involved comparing the largest spatial correlation between a patient's frame and the CAPs to the distribution of spatial correlations of frames from healthy controls belonging to the best matching CAP. A threshold at the 5th percentile was set to determine the best match.^34^ A more detailed description of the procedure can be found by Weber et al.^13^

**FIGURE 1:**
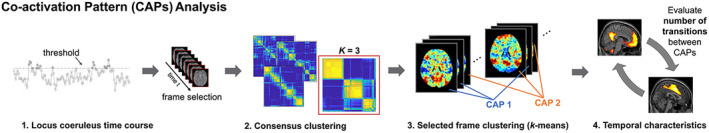
Co‐activation pattern (CAP) analysis. CAP maps were calculated using the locus coeruleus (LC) seed. (2) Consensus clustering was performed on in order to identify the most stable cluster. (3) The fMRI were volumes selected by LC activation were clustered in four states using k‐means algorithm. (4) Number of entries into states, transition probabilities between CAPs, and duration of CAPs across the time‐course were computed for each subject.

We quantified the significant voxels laying within each CAP by computing their overlap with the Yeo network atlas (in %).^37^ Lastly, we evaluated the temporal characteristics of each CAP for which we calculated the number of entries into each specific CAP (“Entries”), the average duration of the individual CAPs (“Duration,” i.e., the average number of sequential volumes assigned to a CAP multiplied by the relaxation time [TR]), and the transition probability (i.e., the probability to enter a certain CAP given being in a certain other or same CAP). As such, we calculated the transition probability matrix *T* for each subject, which recapitulates the probability to switch from one CAP at timepoint *t* to another CAP at timepoint *t* + 1. Significant differences in transition probabilities were assessed using multiple *t*‐tests, corrected for false discovery rate (FDR) at a significance level of *p* < *α*, at which the *α*‐level corresponded to 0.05. Lastly, temporal characteristics in patients were correlated to the number of past seizures. All analyses were corrected for the number of excluded fMRI frames (derived from FD), age, sex, psychotropic medication intake, depression (BDI score), and state anxiety (STAI‐S score). Furthermore, statistical analyses were corrected for multiple comparisons. As a control analysis, we repeated all calculations using the red nucleus as a seed region. The red nucleus was chosen as it is of comparable volume to the LC and is also a subcortical nucleus in relative proximity to the LC, without being part of the ascending arousal network of brainstem nuclei. A visual representation of the CAPs methodology can be found in Figure 1. The code used for these analyses is available under https://github.com/webersamantha/PCA_CAP_SW.

## 3 RESULTS

### 3.1 Clinical and demographic characteristics

Patients with dissociative seizures reported higher numbers of depressive symptoms compared to healthy controls but reported the same level of state anxiety on average (Table 1).

Patients differed from controls in number of excluded frames based on Power's framewise displacement criterion^33^ with more excluded volumes in patients. However, they did not significantly differ from controls in the number of selected frames for the CAP analyses, which indicates that despite scrubbing, equal numbers of functional volumes were selected across the two groups. Demographic and clinical data are presented in Table 1.

### 3.2 Locus coeruleus co‐activation patterns

**FIGURE 2:**
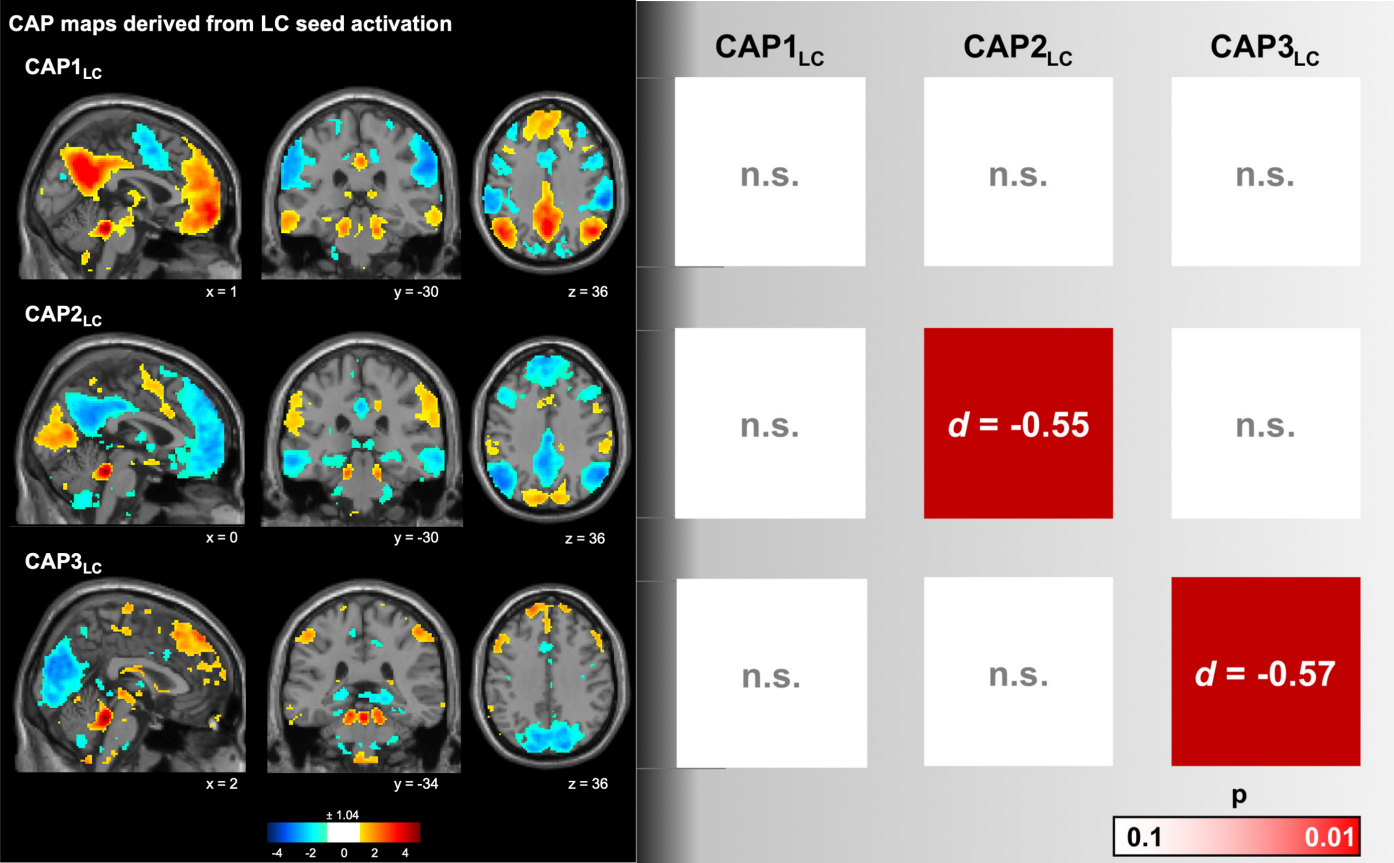
Co‐activation pattern (CAP) maps based on locus coeruleus seed activation. Three CAPs were identified, and their contributions were standardized using z‐scores. In the visual representation, only the top 15% most positively and negatively contributing components are displayed (*z*‐scores of ±1.04). Positive contributions are depicted in red, while negative contributions are shown in blue. The spatial coordinates are displayed in Montreal Neurological Institute (MNI) standard space. Effect sizes of the transition probabilities between CAPs are depicted. The transition probability matrix *T*_*m,n*_ represents the probability of moving from one state *m* to another state *n*, PR(n|m) = *T*_*m,n*_.

**FIGURE 3:**
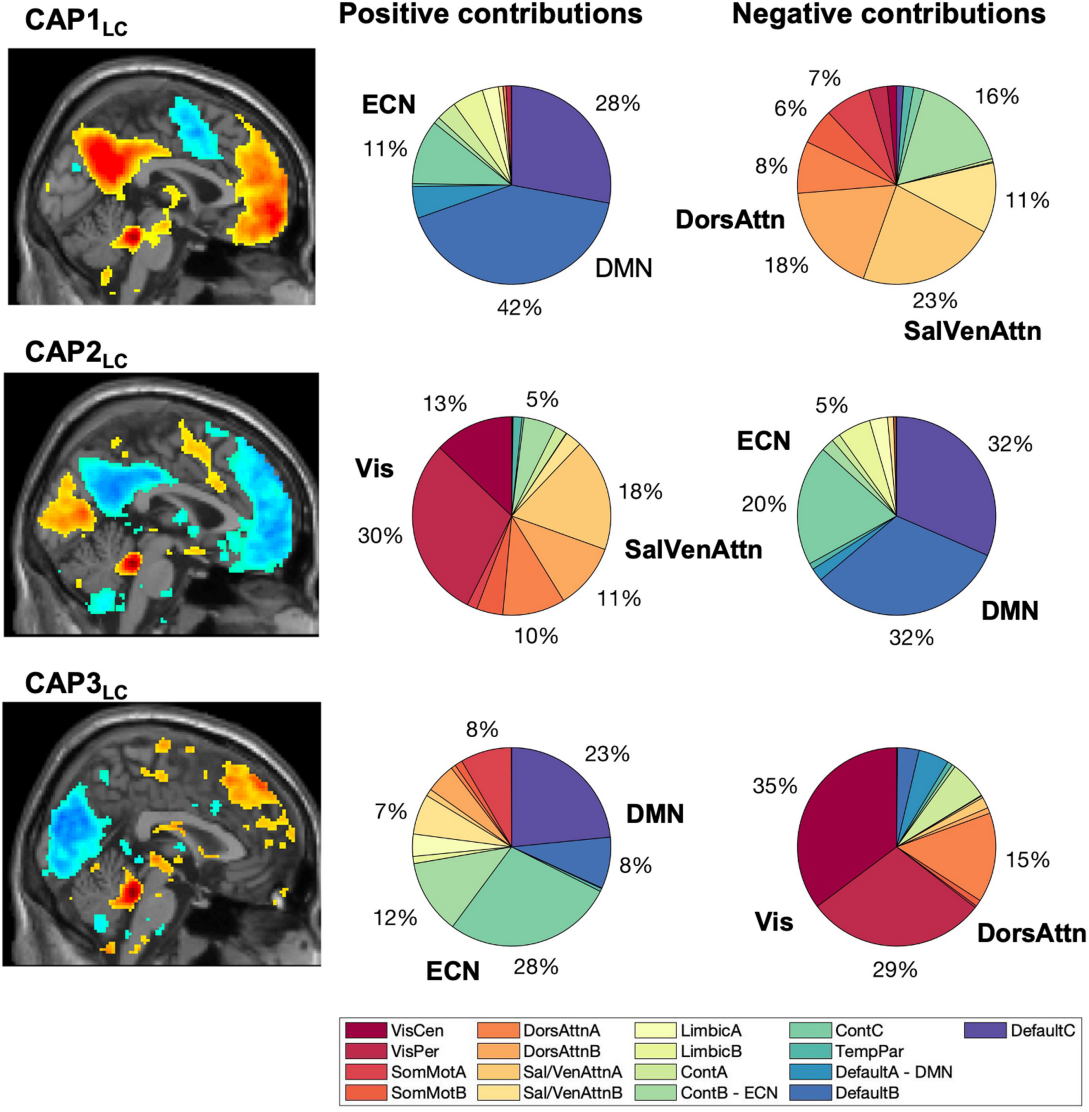
Spatial overlap (in %) of CAPs with functional network atlas. The pie charts provide an overview of the distribution of positive and negative contributions within the 17 resting‐state networks, following the Yeo convention. Seed voxels were removed. LC, locus coeruleus; HC, healthy controls; Cont, executive control; Default, default mode DorsAttn, dorsal attention; Sal/VenAttn, salience/ventral attention; SomMot, somatomotor; TempPar, temporoparietal; VisCen, central vision; VisPer, peripheral visual.

On average, 60.6 frames were selected in the healthy control population, of which on average 7.04 (±5.5) frames remained unassigned. In the PNES group, on average 56.9 frames were selected of which 13.4 (±13.09) frames remained unassigned. Three CAPs corresponding to locus coeruleus co‐activation were identified (Table 2, Figure 2). The first CAP (CAP1_LC_) exhibits a LC co‐activation predominantly with the default mode network, and regions/nuclei such as the substantia nigra, the mediodorsal thalamic nucleus, and the hippocampus; and a co‐deactivation with the dorsal (DorsAttn) and salience/ventral attention networks (Sal/VenAttn), spanning regions such as the supplementary motor area (SMA), the insula, the precentral gyrus, and the supramarginal gyrus. The second CAP (CAP2_LC_) denotes an activation pattern with co‐activation of the visual network (Vis) and the Sal/VenAttn, and regions including the SMA, the insula, and the supramarginal gyrus; and a co‐deactivation with the default mode network and the executive control network, and regions such as the hippocampus, the anterior nucleus of the thalamus, and the head of the nucleus caudate. The third CAP (CAP3_LC_) represents a LC activation pattern with co‐activation of the executive control network and default mode network, covering regions such as the hippocampus and parahippocampal regions, and the inferior parietal lobule; as well as co‐deactivation of the Vis and DorsAttn (Figure 3). Most functional volumes were assigned to CAP1_LC_ (39.16%), 37.06% of the volumes were assigned to CAP2_LC_ and 23.78% to CAP3_LC_.

**TABLE 2:** AAL3‐defined regions contributing to CAPs.

	Region positive contribution [MNI coordinates]	Regions negative contribution [MNI coordinates]
CAP1_LC_	Angular gyrus [±45, −61, −30] Precuneus [0, −54, −28] Mid cingulate cortex [0, −30, −35] Superior medial frontal gyrus [0, 55, 18] Medial orbital frontal gyrus [0, 60, −10] Gyrus rectus [0, 44, −18] Midtemporal gyrus [±64, −4, −25]	Supramarginal gyrus [±62, −30, 30] Inferior Frontal Gyrus [±58, 10, 6] Insula [±46, 9, −3]
CAP2_LC_	Calcarine [0, −80, 10] Precuneus [0, −54, −28] Mid cingulate cortex [0, 20, 32] Supplementary Motor Area [±5, 6, 52] Inferior Frontal Gyrus [±58, 10, 6] Insula [±46, 9, −3]	Cerebellum [±40, −68, −40] Angular gyrus [±45, −61, −30] Middle frontal gyrus [±40, 20, 46] Temporal pole [±40, 18, −40] Ncl. Accumbens [±10, 18, −5]
CAP3_LC_	Middle frontal gyrus [±40, 20, 46] Inferior parietal cortex [±40, −55, 50] Insula [±46, 9, −3]	Fusiform gyrus [±42, −70, −15] Calcarine [0, −80, 10] Anterior cingulate cortex [0, 18, 30]

**FIGURE 4:**
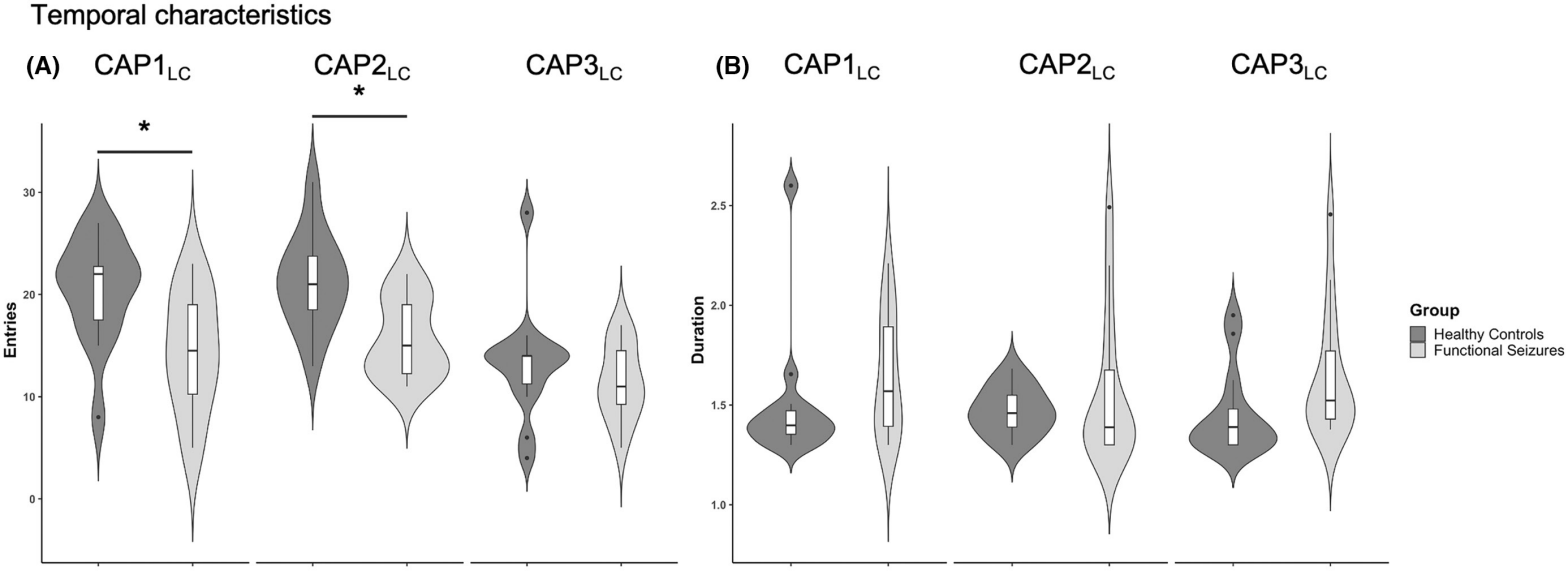
Co‐activation pattern (CAP) temporal measures. For CAPs derived from healthy controls compared to patients with dissociative seizures numbers of entries (right) and duration (left). Asterisks indicate statistical significance for data adjusted for covariates (i.e., number of excluded fMRI frames [derived from FD], number of selected frames [derived from CAPs], age, sex, psychotropic medication [dichotomous], depression [BDI], and anxiety [STAI‐S] scores) and corrected for multiple comparisons with *p < 0.05, **p < 0.01, ***p < 0.001. Boxplots: Horizontal lines represent group median; box represents interquartile range, and vertical line represents 1.5‐times interquartile range. Violin plots visualize the distribution of the data. BDI, Beck's Depression Inventory‐II; STAI, State–Trait Anxiety Inventory.

Adjusted for age, sex, number of discarded volumes, number of selected volumes, psychotropic medication, and depression and anxiety scores, group comparisons of temporal characteristics revealed that patients with dissociative seizures entered less often into CAP1_LC_ (*P*_FDR_ = 0.0023) as well as less often into CAP2_LC_ (*P*_FDR_ = 0.023) than healthy controls (Figure 4A). Regarding average CAP durations, no significant between‐group differences were identified (Figure 4B).

The analysis of state transition probabilities (Figure 3 and Table S1) revealed higher transition probabilities from CAP2_LC_ to CAP2_LC_ as well as from CAP3_LC_ to CAP3_LC_, that is, a higher probability of remaining in CAP2_LC_ or CAP3_LC_, respectively (state persistence), as compared to healthy controls (both *P*_FDR_ = 0.01). CAP's temporal characteristics in patients did not correlate with number of seizures within the past 4 weeks.

To assess the specificity of this finding, we performed a control condition analysis using the red nucleus as a seed region. There was no significant between‐group difference for any of the transition probabilities (see Supplementary Material).

## 4 DISCUSSION

Current models of dissociation and of dissociative seizures suggest that the temporary loss of integrative functions (such as self‐awareness and behavioral control) in the context of abnormal affective arousal might be related to specific alterations in network dynamics.^9^, ^38^, ^39^, ^40^ In our analysis of resting‐state fMRI, two of three identified LC‐CAPs showed higher state persistence rates compared to healthy controls. This finding was adjusted for state anxiety and depression scores, and was not seen in CAPs seeded from a similarly sized midbrain nucleus, the red nucleus. This offers a reasonable degree of pathophysiological relevance to the occurrence of dissociative seizures. We suggest that our findings, although limited in their interpretability by the relatively small sample size, provide first evidence that arousal‐mediated dissociation might be related to a temporary impairment in network switching.

During dissociative seizures, patients with sustained awareness commonly report an inability to interact adequately with their environment, that is, to initiate and execute goal‐directed behavior. Akinetic manifestations are common and have been linked to innate stress‐related freezing behavior. Patients with reported loss of (self‐)awareness, on the other hand, are often observed to remain in a state of continuous erratic motor activity, which has elements of defensive behaviors such as thrashing movements, body rocking, and opisthotonus. These types of dissociative states could be related (if not attributed) to an impairment in flexibility of switching between the default mode and attentional brain networks. In this light, our finding of increased LC‐activity‐related “state persistence” among two out of three CAPs could substantiate the presumed link between central noradrenergic activation and dissociation.

Previous studies in dissociation and functional neurological disorder have identified alterations in the default mode network with clinical correlations. Functional connectivity strength within the default mode network has been found to relate to dissociation scores,^10^ and cortical thickness of default mode network nodes negatively correlated with longer illness duration.^41^ Gallucci‐Neto et al. (2021) found increased connectivity within the default mode network during dissociative seizures.^11^ In children with functional neurological disorder, electroencephalography (EEG) studies indicate aberrant power spectra in default mode areas suggesting chronic overactivation.^42^ These findings are also interesting because the default mode network as a domain‐general network that—insulated from direct interaction with the external world—is responsible for the integration of external and internal information in a “perceptually‐decoupled state.”^43^ Within predictive processing frameworks, it entails abstract concepts that form the basis for probabilistic predictions, which are assumed to be altered in functional neurological disorder.^39^, ^44^ Fittingly, in a previous study from our group also using the CAP approach in an overlapping, larger sample of patients with mixed functional neurological disorder, we found co‐deactivation of the bilateral insulae (the primary interoceptive cortices) with the default mode network.^13^ We interpreted this as dysfunctional bottom‐up integration of interoceptive signals. Together with our current findings, this is in line with the idea of aberrant somatosensory integration and bodily dissociation in functional neurological disorder, especially during states of heightened arousal.^39^, ^45^

Our small exploratory study has limitations. According to standard procedures in the area, the optimal cluster size was based on visual inspection of the consensus matrices and based on the stability measures derived from consensus clustering. While this might be common practice, it is still susceptible to a suboptimal selection of the final cluster. Moreover, using the LC as a seed region is susceptible to noise due to its small size and proximity to the fourth ventricle. Likewise, a significant concern is the delineation of the LC mask. The translation from the 7T LC mask to our population's standard space might introduce potential inaccuracies, particularly when accounting for factors such as smoothing, motion, cerebrospinal fluid pulse, and susceptibility artifacts. Even though the authors state that the atlas does not require 7T or similar sequences,^35^ manual delineation methods might be considered for validation purposes in larger proof‐of‐concept studies.

Patients were found to have higher framewise displacement, for which scrubbing was applied to minimize the deleterious effects of motion artifacts. While this process allows for computing clean CAPs, it also carries the risk to distort transition probability estimates.^34^ Moreover, not all patients were free from psychotropic medication intake, which might influence functional alterations in the brain independent of the disorder itself. However, we corrected our analyses for a potential effect arising from depression, anxiety, or intake of psychotropic medication, as well as performed scrubbing. Our small sample size and the heterogeneity of our patient group further limits the interpretability of our results, while it must be mentioned that this study was designed as a pilot, exploratory study paving the way for a prospective study with larger sample. Ideally, in a larger sample comorbid anxiety disorders can be better controlled for. Lastly, the observed temporal alterations in patients did not correlate with number of experienced seizures within the past 4 weeks.

## 5 CONCLUSION

Our analysis of resting‐state fMRI from patients with dissociative seizures reveals group‐level differences in the transition probabilities of LC‐CAPs compared to healthy controls subjects. Increases in state persistence across two of three identified CAPs suggest that central noradrenergic activation might impair network switching flexibility to a degree that could critically disrupt (self‐)awareness and behavioral control during dysregulated affective arousal. This hypothesis should be further explored with other neuroimaging modalities and for other forms of dissociation including experimentally induced phenomena. Lastly, if the role of the central noradrenergic system is further corroborated, pharmacological targeting might become clinically viable.

## AUTHOR CONTRIBUTIONS

Samantha Weber: Data curation, formal analysis, methodology, visualization, writing—original draft, and writing—review and editing. Johannes Jungilligens: Conceptualization, writing—original draft, and writing—review and editing. Selma Aybek: Funding acquisition, supervision, and resources. Stoyan Popkirov: Conceptualization, writing—original draft, and writing—review and editing.

## FUNDING INFORMATION

This work was supported by the Swiss National Science Foundation (SNF grant PP00P3_176985) and the University Hospital Inselspital Bern, Switzerland. SP is supported by a BMBF Advanced Clinician Scientist Programme UMEA2 (01EO2104).

## CONFLICT OF INTEREST STATEMENT

None of the authors has any conflict of interest to disclose.

## Supporting information


Data S1.

## Data Availability

The data are not publicly available due to restrictions demanded by the administering institution to guarantee the privacy of the participants. The data can be shared upon request.
