# Supplementary material for: Locus coeruleus co‐activation patterns at rest show higher state persistence in patients with dissociative seizures: A Pilot Study
Source: Epilepsia Open. 2024 Oct 7;9(6):2331–41. doi: 10.1002/epi4.13050 (PMC11633765; doi:10.1002/epi4.13050)
Supplement: Supplementary file 1 — Data S1. [file EPI4-9-2331-s001.docx]

**Locus coeruleus co-activation patterns at rest show higher state persistence in patients with dissociative seizures: A Pilot Study**

**Samantha Weber*** ([samantha.weber@unibe.ch](mailto:samantha.weber@unibe.ch), ORCID: 0000-0002-2689-2938)

Department of Neurology, Psychosomatic Medicine Unit, Inselspital Bern University Hospital, University of Bern, 3012 Bern, Switzerland

University of Zurich, Psychiatric University Hospital Zurich, Department of Psychiatry, Psychotherapy and Psychosomatics, 8032 Zurich, Switzerland.

Translational Imaging Center (TIC), Swiss Institute for Translational and Entrepreneurial Medicine, 3010 Bern, Switzerland.

**Johannes Jungilligens*** ([johannes.jungilligens@rub.de](mailto:johannes.jungilligens@rub.de); ORCID: 0000-0002-4846-8337)

Department of Neurology, University Hospital Knappschaftskrankenhaus, Ruhr University Bochum, Bochum, Germany

**Selma Aybek^+^** ([selma.aybek@unifri.ch](mailto:selma.aybek@unifri.ch); ORCID: 0000-0002-7877-6760)

Faculty of Science and Medicine, University of Fribourg, 1700 Fribourg, Switzerland

Department of Neurology, Psychosomatic Medicine Unit, Inselspital Bern University Hospital, University of Bern, 3012 Bern, Switzerland

**Stoyan Popkirov^+^** ([stoyan.popkirov@uk-essen.de](mailto:stoyan.popkirov@uk-essen.de); ORCID: 0000-0001-6168-0036)

Department of Neurology, University Hospital Essen, Hufelandstr. 55, 45147 Essen, Germany

*SW and JJ contributed equally to this work.

**^+^**SA and SP contributed equally to this work.

**LC CAPs derived from HC**

**Supplementary Figure 1. Stability measure (1 – PAC).** To determine the stability of a particular cluster number, it's essential to observe if two specific data points consistently belong to the same cluster or different clusters in multiple iterations. Calculating the cumulative distribution of consensus values for all pairs of data points provides a quantitative measure of how well the data fits the clustering model. This distribution is referred to as $P_{k}\left( c \right) with c\in\left[ 0,1 \right]$. From this, the proportion of ambiguously clustered pairs (PAC) can be calculated^1^ as $PAC_{k}=\sum_{C=C_{T}}^{1-C_{T}} P_{k}\left( c \right)$, with *c_T_* a threshold consensus value that, when exceeded, indicates that an assignment is considered insufficiently uniform or consistent across different iterations, and *k* the cluster number. A reduced *PAC* value signifies a more resilient cluster number. The stability metric is subsequently calculated as 1 - *PAC*, meaning that higher values indicate stronger and more robust clusters. The individual bars (coloured) represent the different choices for the threshold *c_T_.* Three CAPs were preferred over two which is trivial, i.e., as the optimization during k-means clustering aims at minimizing the variance within each cluster while maximizing the variance between clusters. Thus, with *N* = 2, this optimization problem is extremely simple because there are only two means to calculate and adjust in each iterations, making the convergence to the solution fast and simple. Three CAPs were preferred over six or more CAPs based on the consensus matrices (Supplementary Figure 3).


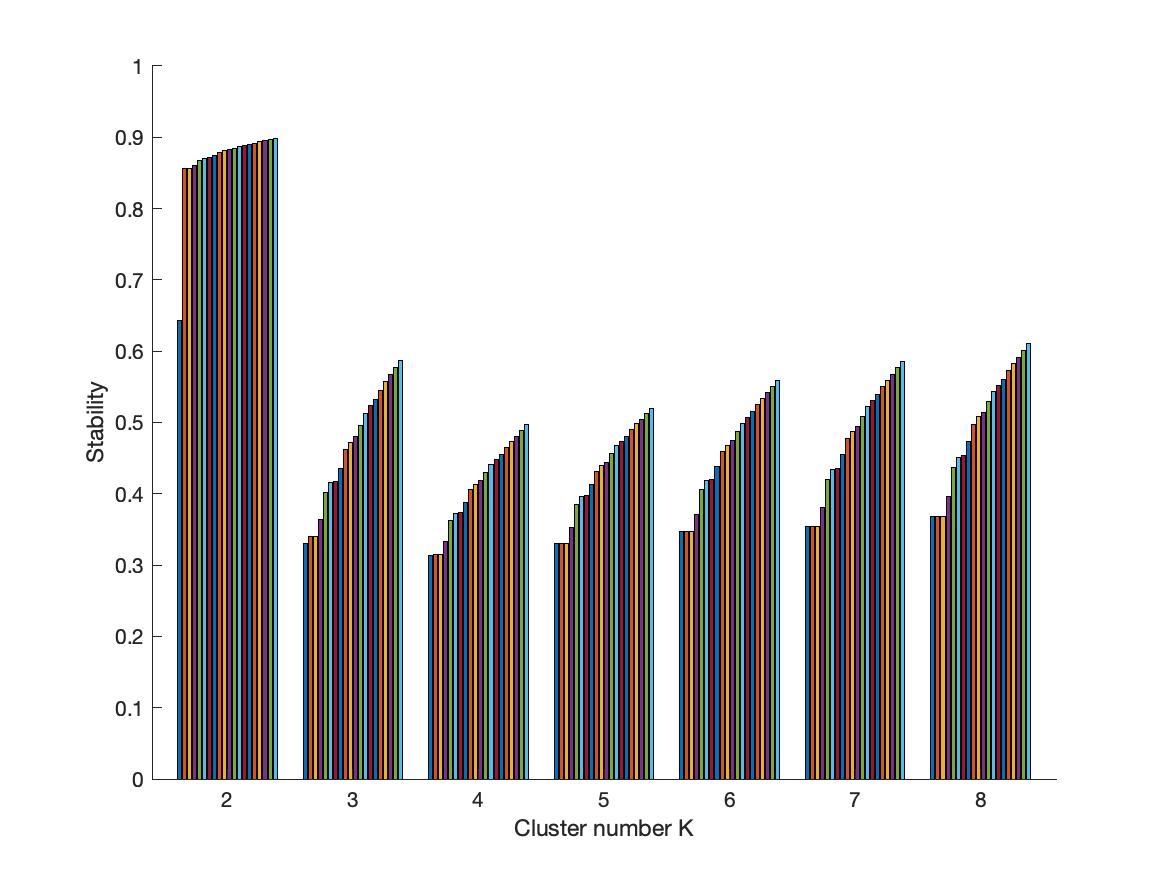


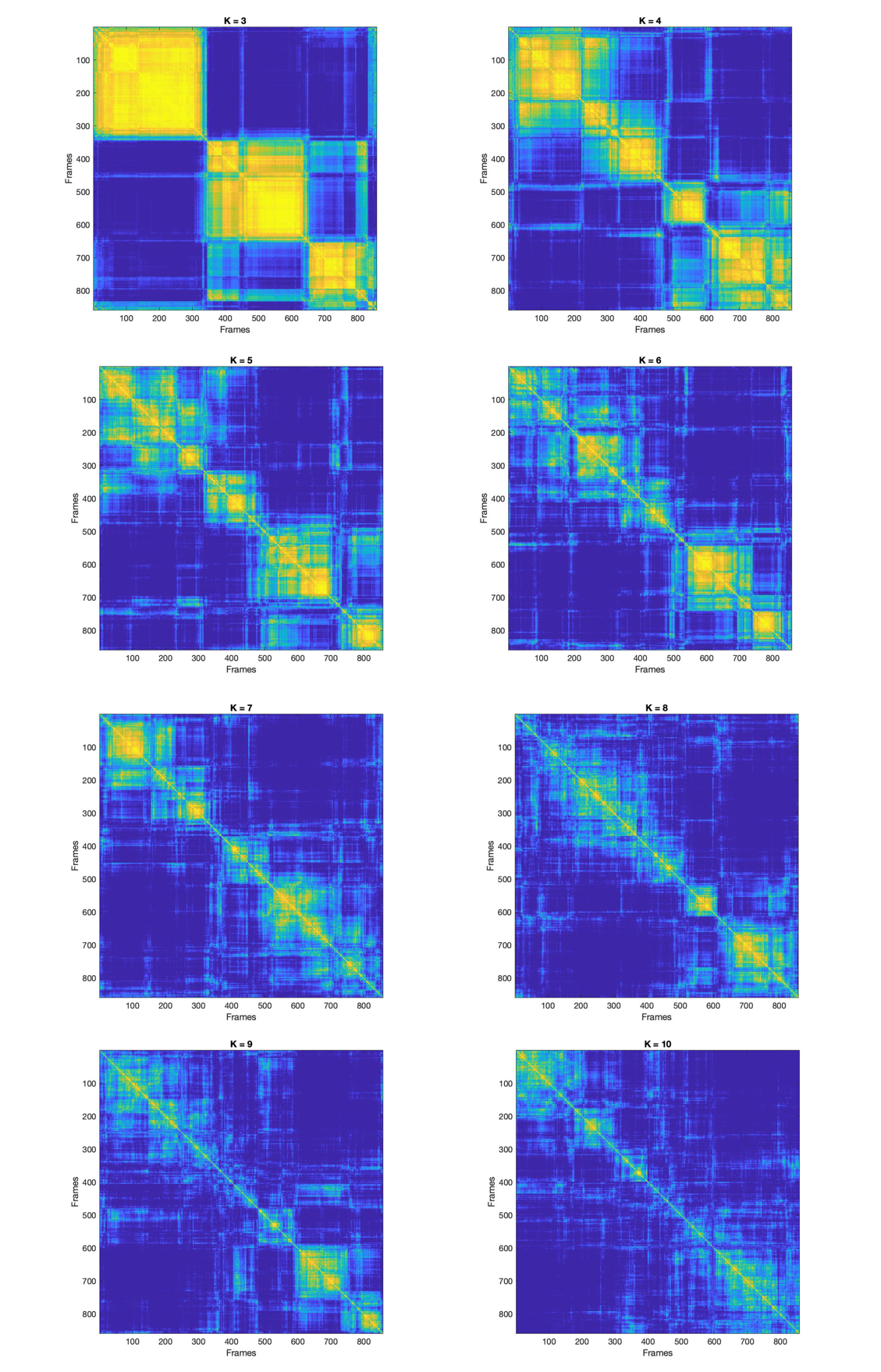
**Supplementary Figure 2. Consensus matrices.** The consensus matrices $C_{k}$, associated with a specific cluster number *k*, serve to condense the consensus values for all possible pairs of data points. They are computed by averaging the values for each entry across all folds where the two data points were jointly involved in the calculations. Cluster stability is achieved when there is a consistent clustering of two random data points, indicated by clear and well-defined boundaries in the consensus matrix.

**Supplementary Table 1. Group differences in transition probabilities.**

|  | **CAP1_LC_** | **CAP2_LC_** | **CAP3_LC_** |
| --- | --- | --- | --- |
| CAP1_LC_ | 0.73 (0) | 0.90 (1) | 0.42 (-0.63) |
| CAP2_LC_ | 0.99 (0.63) | 0.01*(-0.55) | 0.73 (0) |
| CAP3_LC_ | 0.73 (0) | 0.42 (-0.45) | 0.01* (-0.57) |

Significance values (*p*) of group differences between patients with dissociative seizures and healthy controls are shown with effect sizes (Cohen’s *d*) in parentheses. * denotes *p*<0.05.

**LC CAPs derived from dissociative seizures patients.**

**Supplementary Figure 3. Stability measure (1 – PAC).** To determine the stability of a particular cluster number, it's essential to observe if two specific data points consistently belong to the same cluster or different clusters in multiple iterations. Calculating the cumulative distribution of consensus values for all pairs of data points provides a quantitative measure of how well the data fits the clustering model. This distribution is referred to as $P_{k}\left( c \right) with c\in\left[ 0,1 \right]$. From this, the proportion of ambiguously clustered pairs (PAC) can be calculated^1^ as $PAC_{k}=\sum_{C=C_{T}}^{1-C_{T}} P_{k}\left( c \right)$, with *c_T_* a threshold consensus value that, when exceeded, indicates that an assignment is considered insufficiently uniform or consistent across different iterations, and *k* the cluster number. A reduced *PAC* value signifies a more resilient cluster number. The stability metric is subsequently calculated as 1 - *PAC*, meaning that higher values indicate stronger and more robust clusters. The individual bars (coloured) represent the different choices for the threshold *c_T_.* Three CAPs were preferred over two which is trivial.


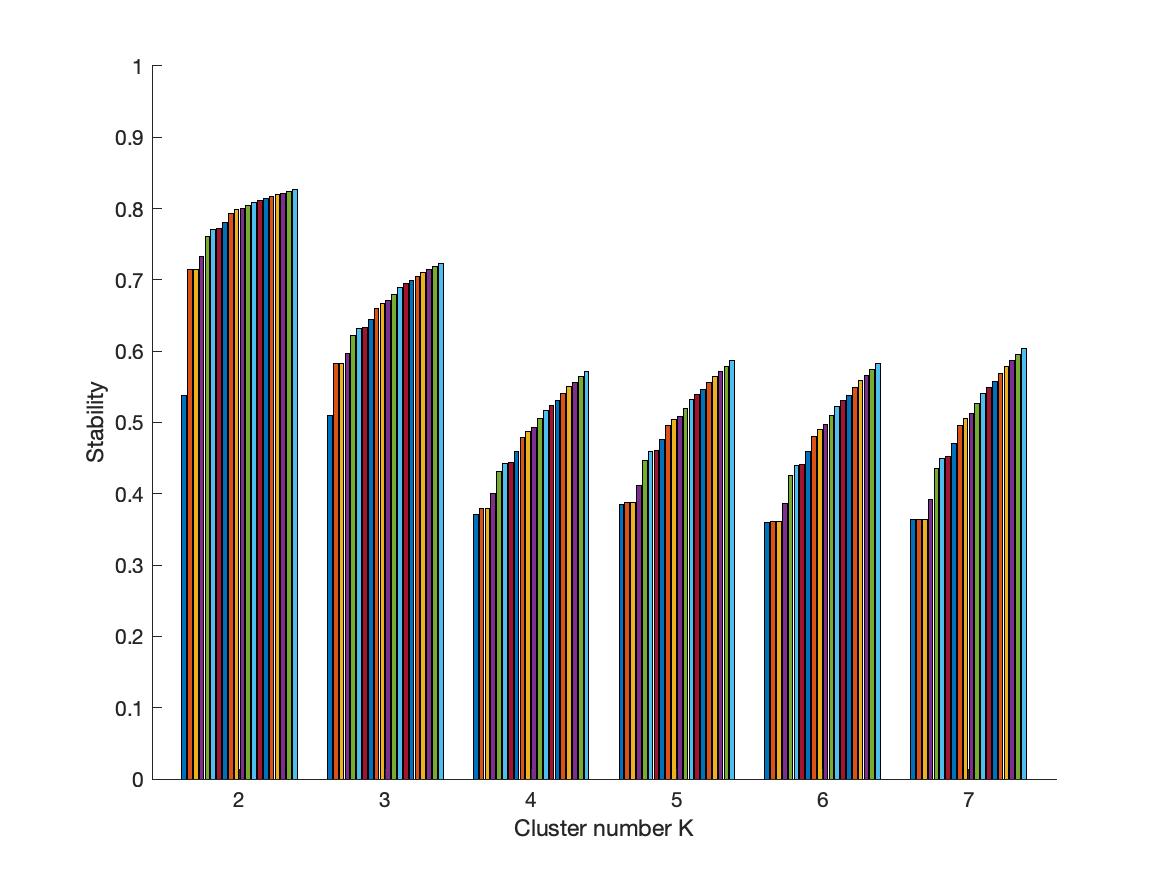


**Supplementary Figure 4. Consensus matrices.** The consensus matrices $C_{k}$, associated with a specific cluster number *k*, serve to condense the consensus values for all possible pairs of data points. They are computed by averaging the values for each entry across all folds where the two data points were jointly involved in the calculations. Cluster stability is achieved when there is a consistent clustering of two random data points, indicated by clear and well-defined boundaries in the consensus matrix.

**
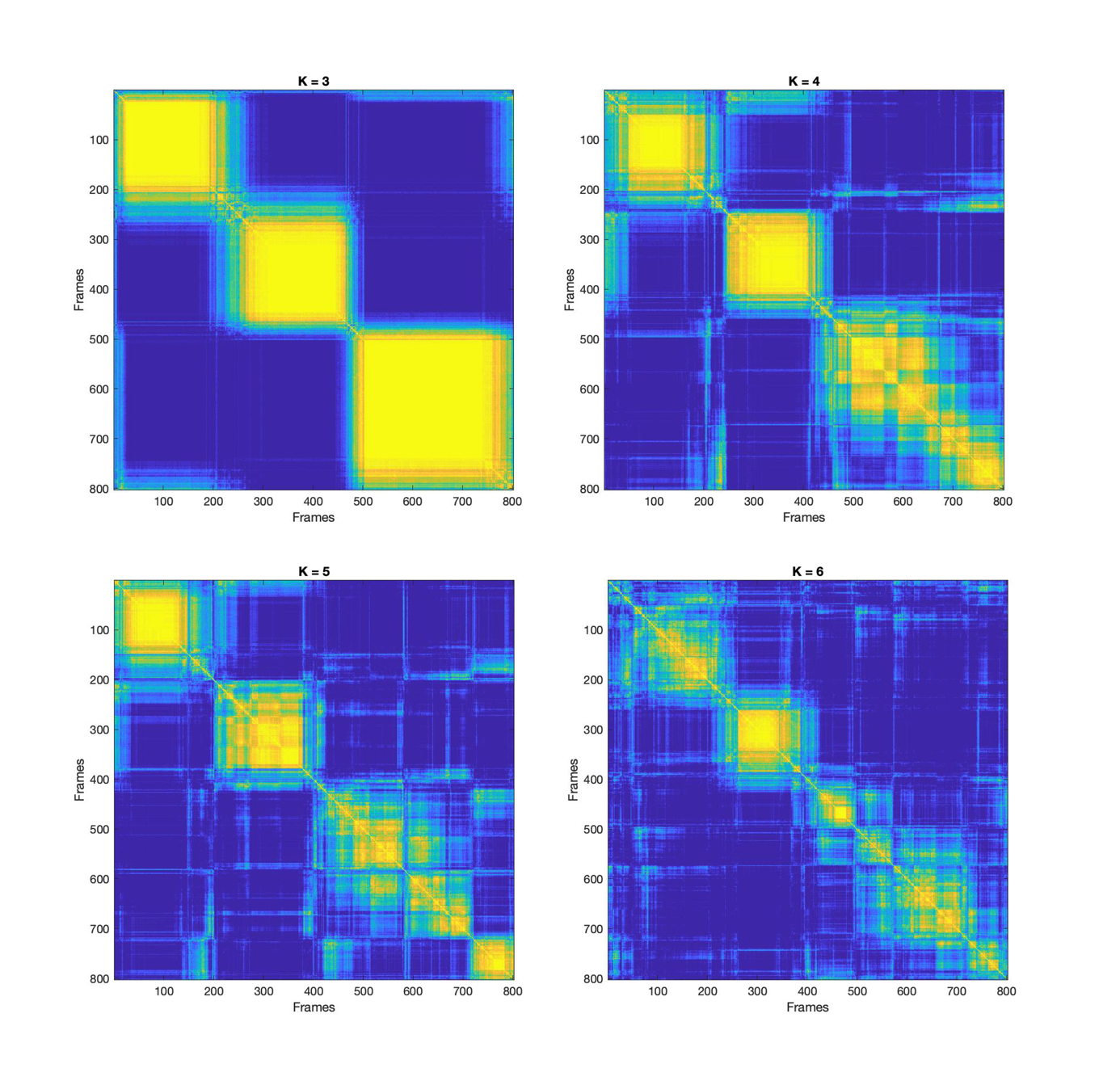
**

**Supplementary Figure 5. Co-activation pattern (CAP) maps based on LC seed activation derived from patients with dissociative seizures** (**A**) Three CAPs were detected. CAPs were z-scored and only the 15% most positive and 15% most negative contributions are represented in colour (z = ± 1.04), with red representing positive contributions and blue negative contributions. Locations are displayed in Montreal Neurological Institute (MNI) standard space coordinates. (**B**) Pie charts illustrating the percentage of positive and negative contributions within the 17 resting-state networks according to the convention of Yeo.^2^ Seed voxels were removed. CAP1_LC_ derived from patients with dissociate seizures overlaps in 86% of the voxels with CAP1_LC_ derived from healthy controls. Likewise, CAP2_LC_ derived from patients overlaps in 83% of the voxels with CAP2_LC_ derived from healthy controls. CAP3_LC_ derived from patients overlaps in 80% of the voxels with CAP3_LC_ derived from healthy controls. Abbreviations: LC = Locus coeruleus, HC = healthy controls, Cont = Executive control, Default = Default mode, DorsAttn = Dorsal attention, Sal/VenAttn = Salience/Ventral attention, SomMot = somatomotor, TempPar = Temporoparietal, VisCen = Central vision, VisPer = Peripheral Visual.


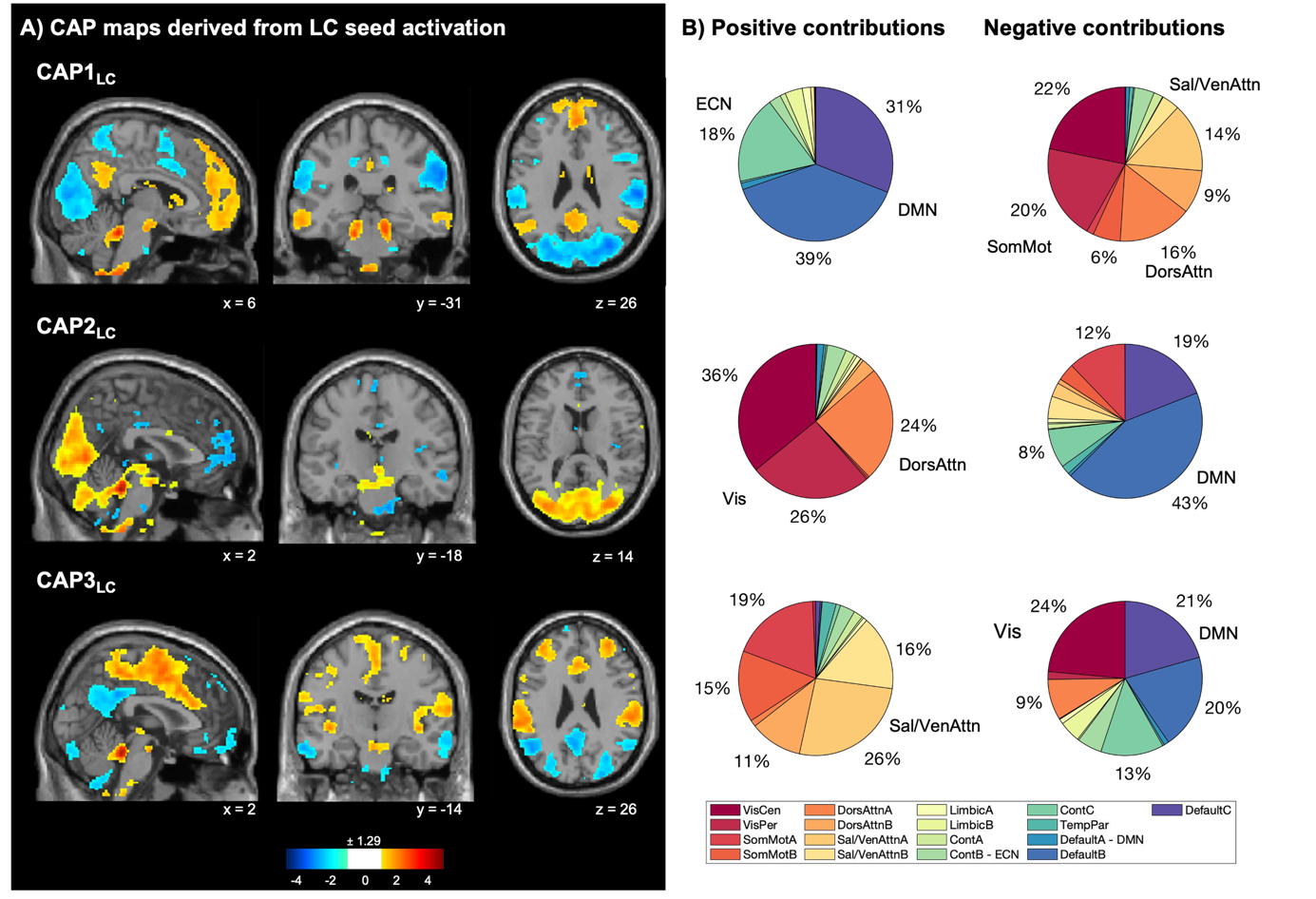


**LC CAPs derived from dissociative seizures patients and healthy controls.**

**Supplementary Figure 6. Stability measure (1 – PAC).** To determine the stability of a particular cluster number, it's essential to observe if two specific data points consistently belong to the same cluster or different clusters in multiple iterations. Calculating the cumulative distribution of consensus values for all pairs of data points provides a quantitative measure of how well the data fits the clustering model. This distribution is referred to as $P_{k}\left( c \right) with c\in\left[ 0,1 \right]$. From this, the proportion of ambiguously clustered pairs (PAC) can be calculated^1^ as $PAC_{k}=\sum_{C=C_{T}}^{1-C_{T}} P_{k}\left( c \right)$, with *c_T_* a threshold consensus value that, when exceeded, indicates that an assignment is considered insufficiently uniform or consistent across different iterations, and *k* the cluster number. A reduced *PAC* value signifies a more resilient cluster number. The stability metric is subsequently calculated as 1 - *PAC*, meaning that higher values indicate stronger and more robust clusters. The individual bars (coloured) represent the different choices for the threshold *c_T_.* Three CAPs were preferred over two which is trivial.

**
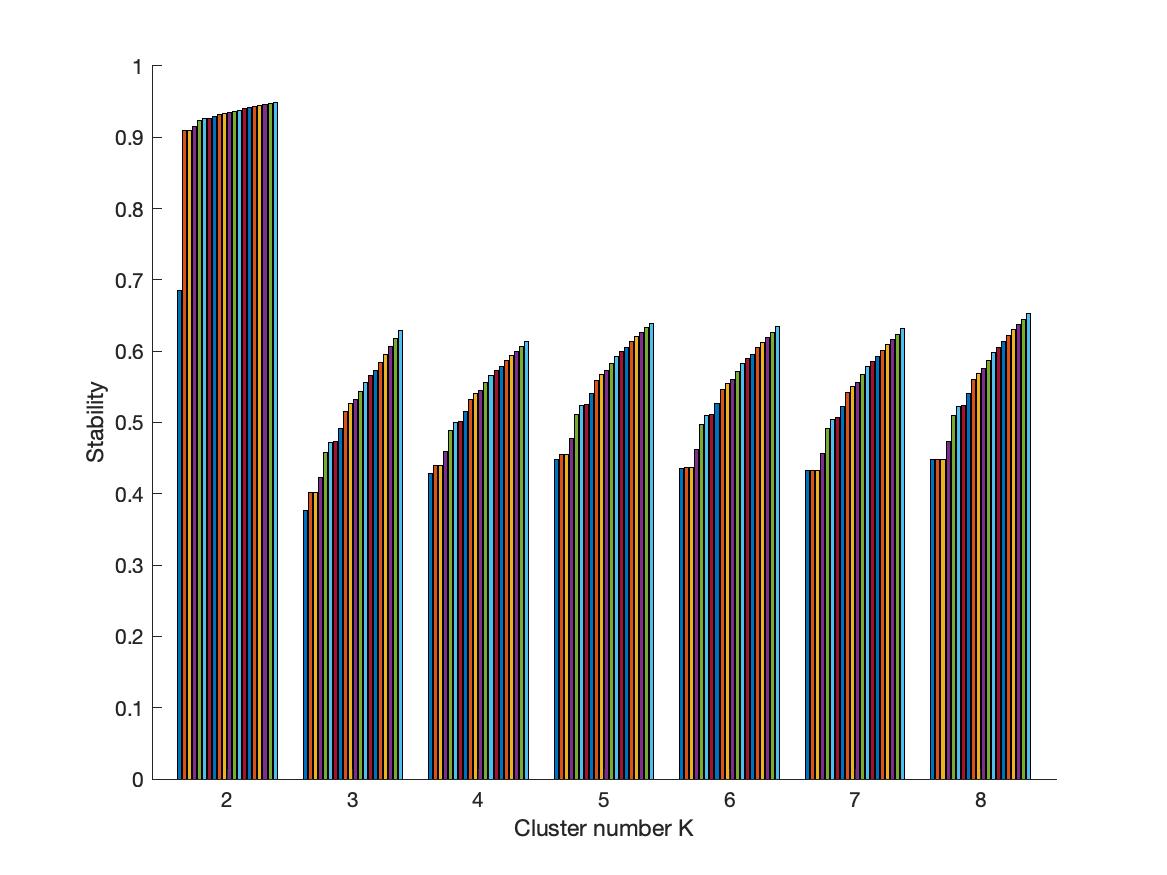
**

**Supplementary Figure 7. Consensus matrices.** The consensus matrices $C_{k}$, associated with a specific cluster number *k*, serve to condense the consensus values for all possible pairs of data points. They are computed by averaging the values for each entry across all folds where the two data points were jointly involved in the calculations. Cluster stability is achieved when there is a consistent clustering of two random data points, indicated by clear and well-defined boundaries in the consensus matrix.


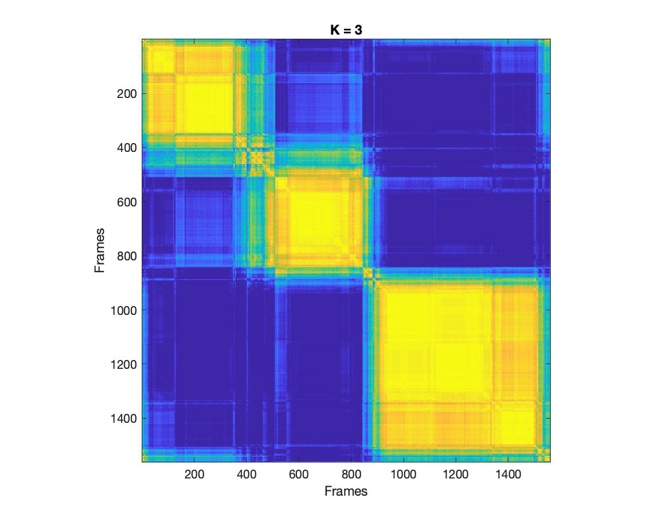

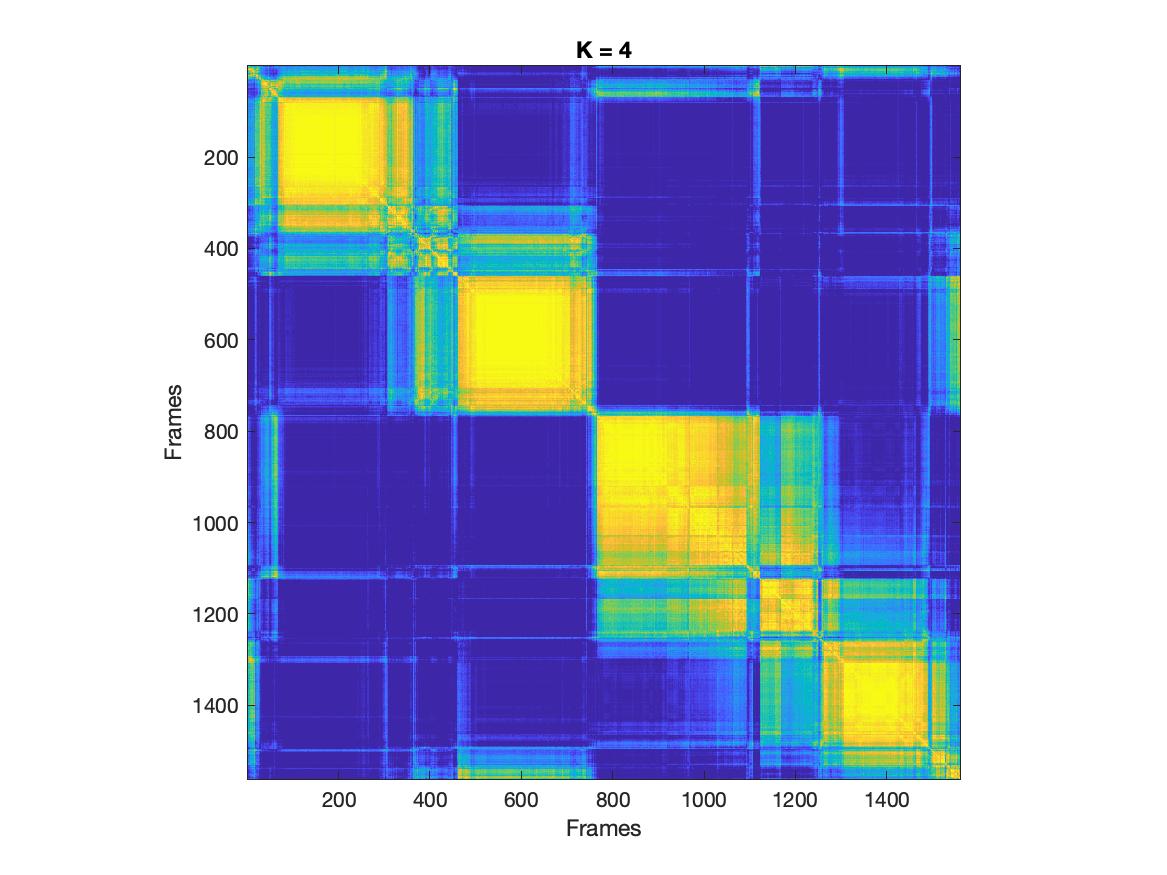

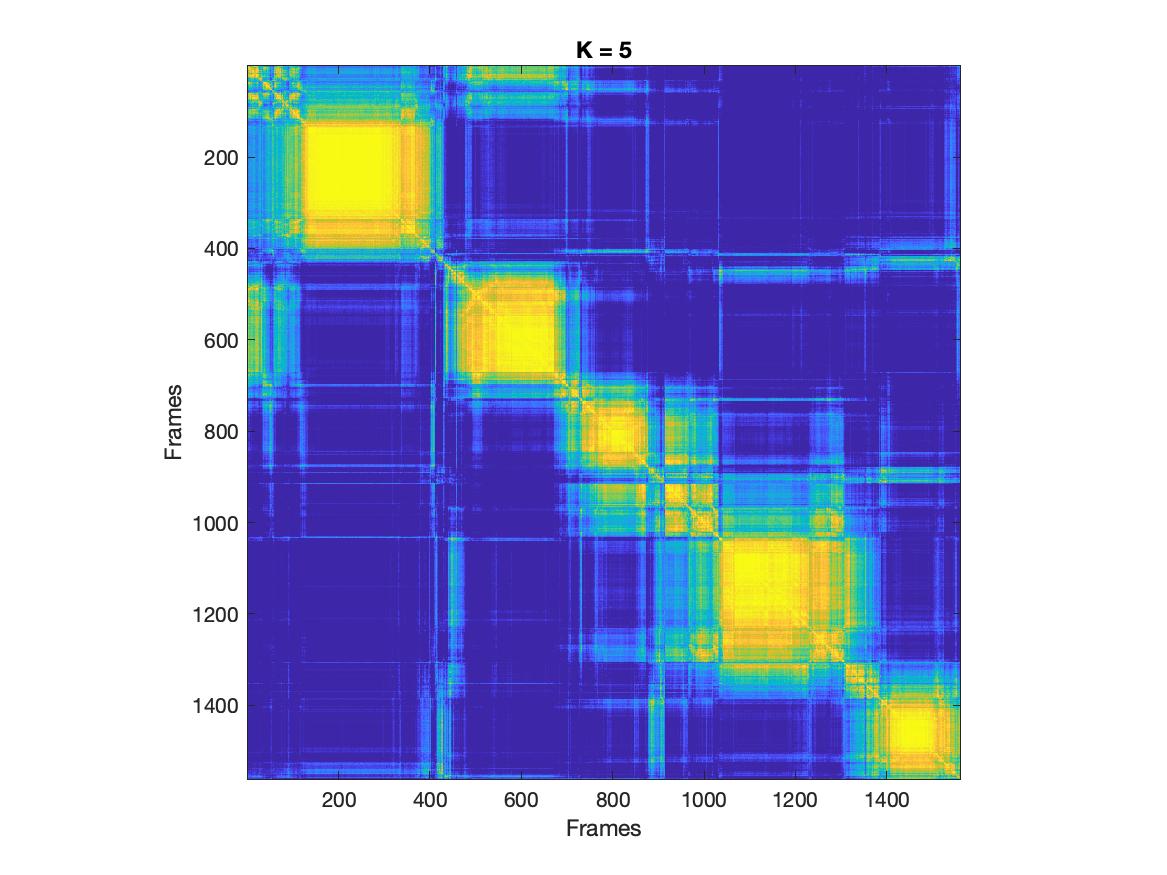

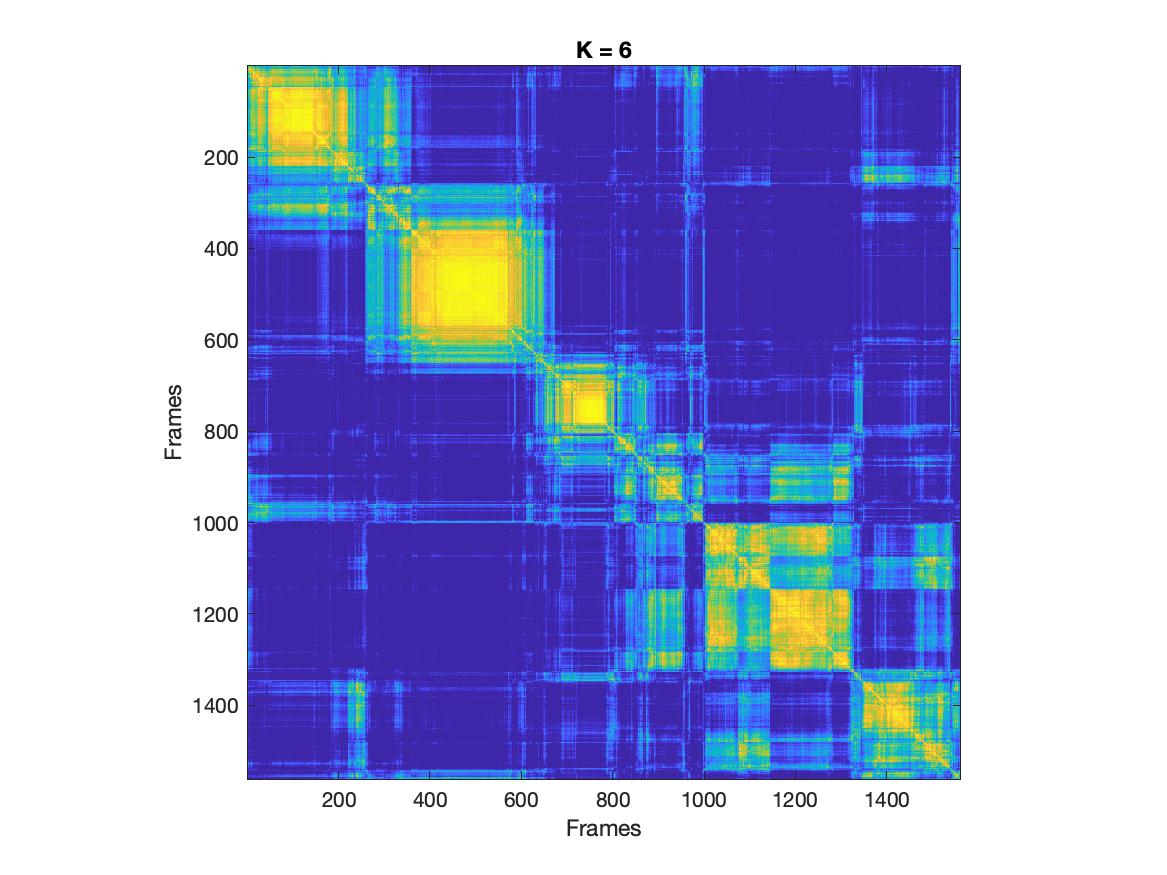

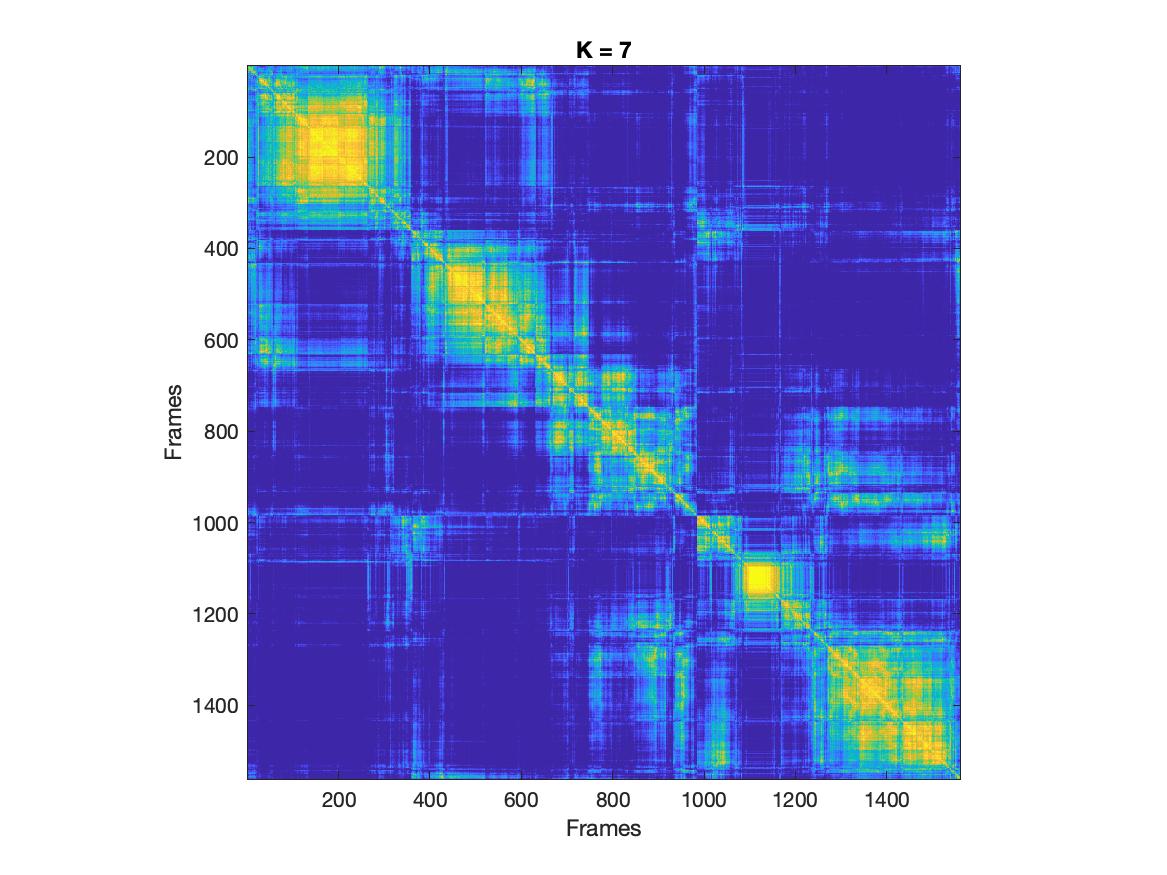

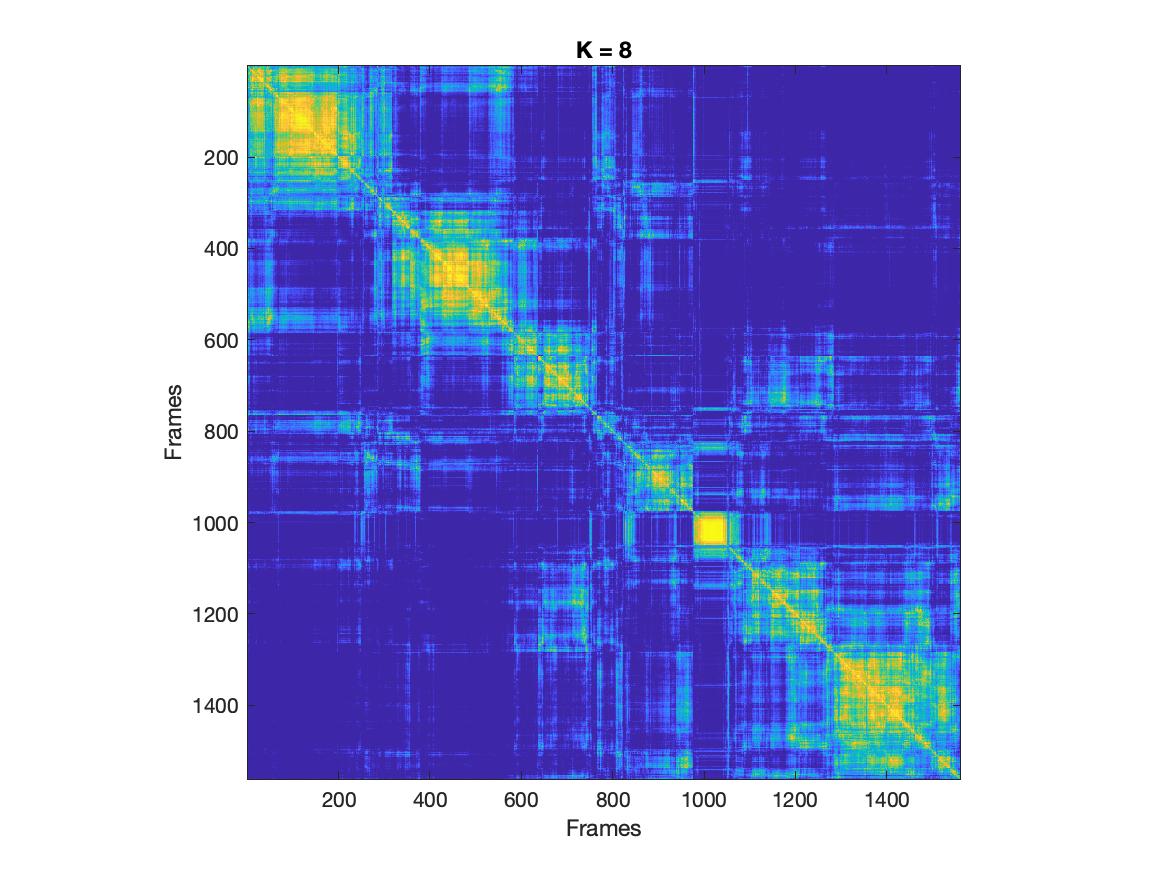


**Supplementary Figure 8. Co-activation pattern (CAP) maps based on LC seed activation derived from patients with dissociative seizures and healthy controls** (**A**) Three CAPs were detected. CAPs were z-scored and only the 15% most positive and 15% most negative contributions are represented in colour (z = ± 1.04), with red representing positive contributions and blue negative contributions. Locations are displayed in Montreal Neurological Institute (MNI) standard space coordinates. (**B**) Pie charts illustrating the percentage of positive and negative contributions within the 17 resting-state networks according to the convention of Yeo.^2^ Seed voxels were removed. CAP1_LC_ derived from patients with dissociate seizures overlaps in 85% of the voxels with CAP1_LC_ derived from healthy controls. Likewise, CAP2_LC_ derived from patients overlaps in 86% of the voxels with CAP2_LC_ derived from healthy controls. CAP3_LC_ derived from patients overlaps in 78% of the voxels with CAP3_LC_ derived from healthy controls. Abbreviations: LC = Locus coeruleus, HC = healthy controls, Cont = Executive control, Default = Default mode, DorsAttn = Dorsal attention, Sal/VenAttn = Salience/Ventral attention, SomMot = somatomotor, TempPar = Temporoparietal, VisCen = Central vision, VisPer = Peripheral Visual.

**
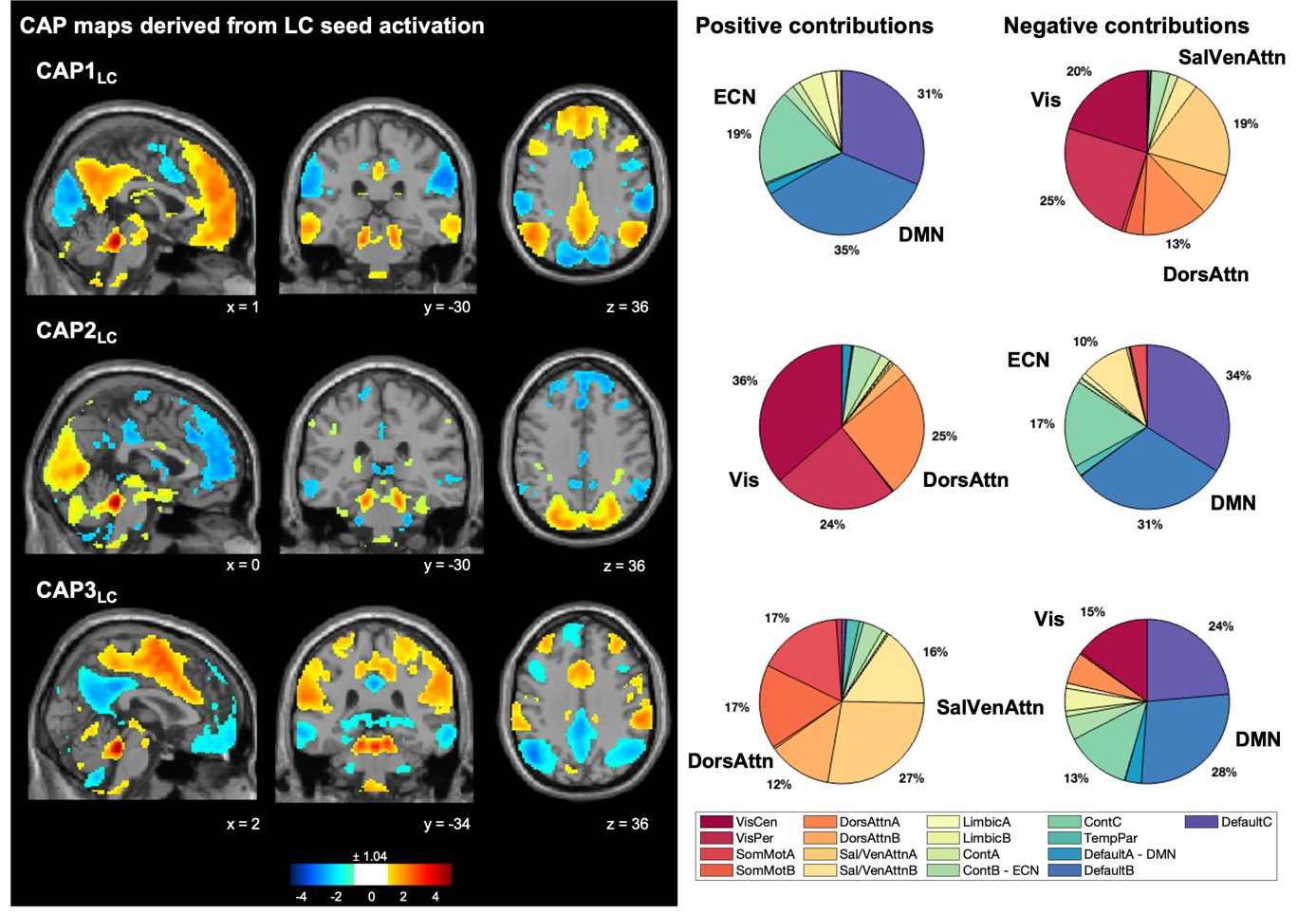
**

**Nucleus ruber CAPs derived from HC as brainstem nuclei control.**

To verify whether our CAPs might have been the result of a spontaneous co-activation with the herein identified networks, we repeated the analyses based on a different (unrelated) brainstem nucleus, i.e., the nucleus ruber. We repeated the analyses using the bilateral Nucleus ruber mask from the Automatic Anatomic Labelling 3 (AAL3) atlas.


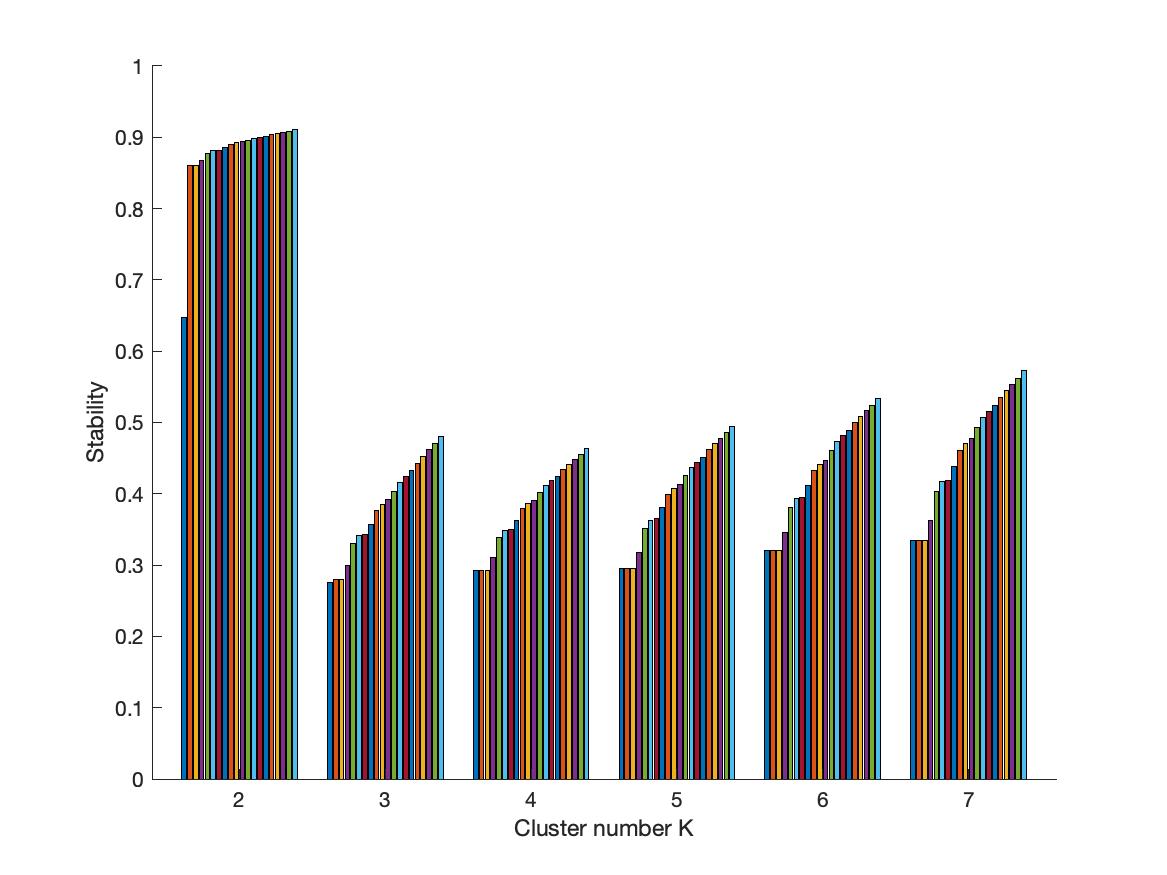
**Supplementary Figure 9. Stability measure (1 – PAC).** To determine the stability of a particular cluster number, it's essential to observe if two specific data points consistently belong to the same cluster or different clusters in multiple iterations. Calculating the cumulative distribution of consensus values for all pairs of data points provides a quantitative measure of how well the data fits the clustering model. This distribution is referred to as $P_{k}\left( c \right) with c\in\left[ 0,1 \right]$. From this, the proportion of ambiguously clustered pairs (PAC) can be calculated^1^ as $PAC_{k}=\sum_{C=C_{T}}^{1-C_{T}} P_{k}\left( c \right)$, with *c_T_* a threshold consensus value that, when exceeded, indicates that an assignment is considered insufficiently uniform or consistent across different iterations, and *k* the cluster number. A reduced *PAC* value signifies a more resilient cluster number. The stability metric is subsequently calculated as 1 - *PAC*, meaning that higher values indicate stronger and more robust clusters. The individual bars (coloured) represent the different choices for the threshold *c_T_.* Three CAPs were preferred over two which is trivial.

**Supplementary Figure 10. Consensus matrices.** The consensus matrices $C_{k}$, associated with a specific cluster number *k*, serve to condense the consensus values for all possible pairs of data points. They are computed by averaging the values for each entry across all folds where the two data points were jointly involved in the calculations. Cluster stability is achieved when there is a consistent clustering of two random data points, indicated by clear and well-defined boundaries in the consensus matrix.


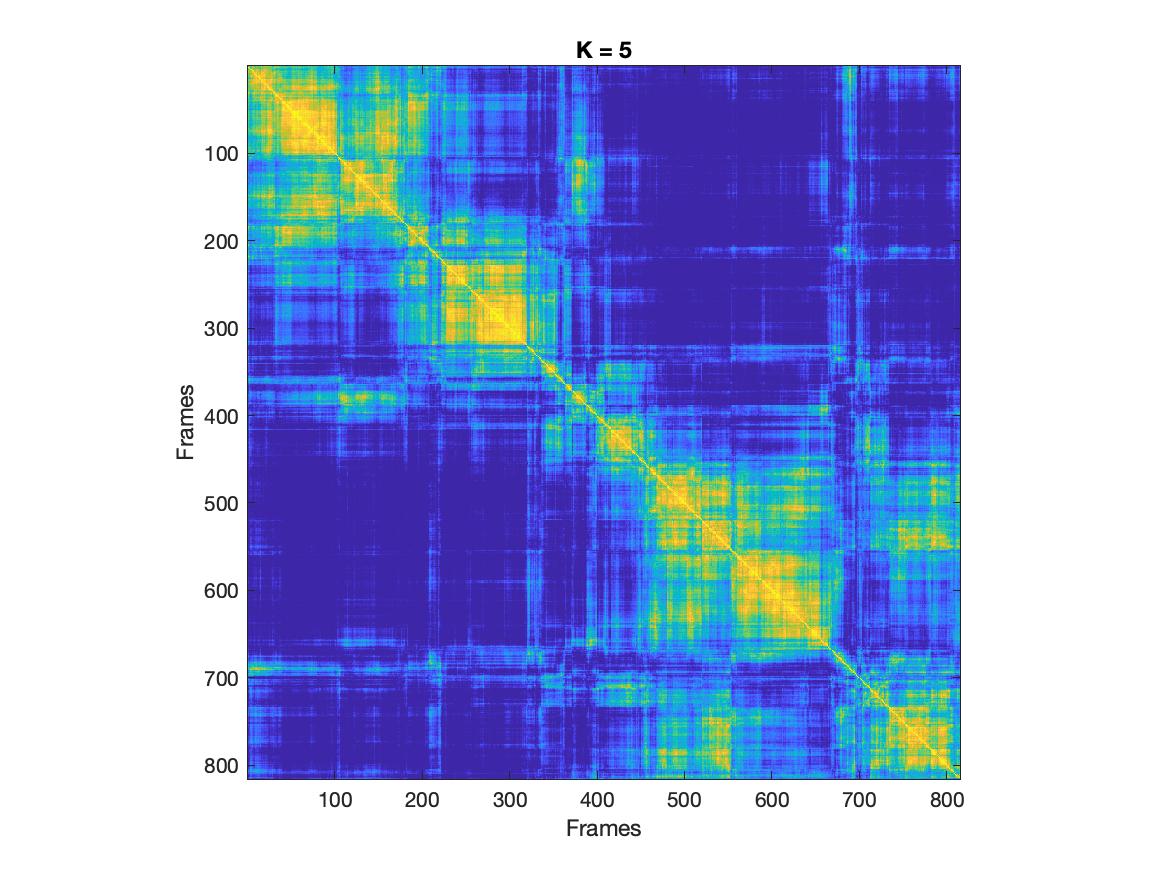

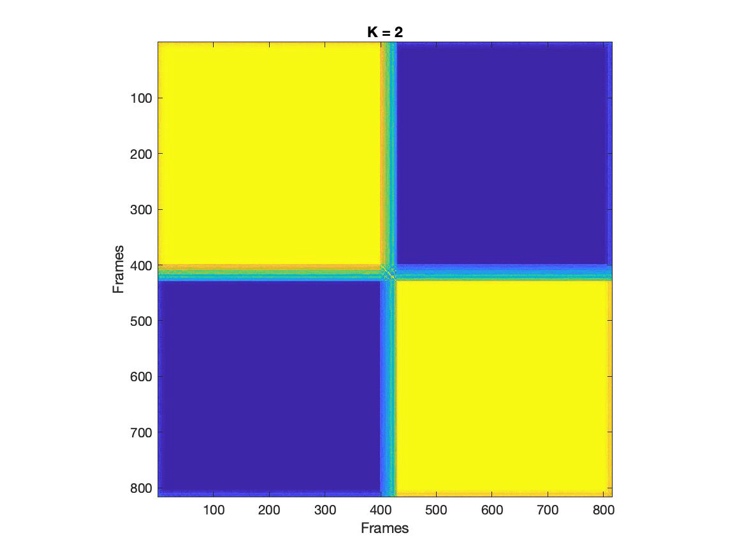

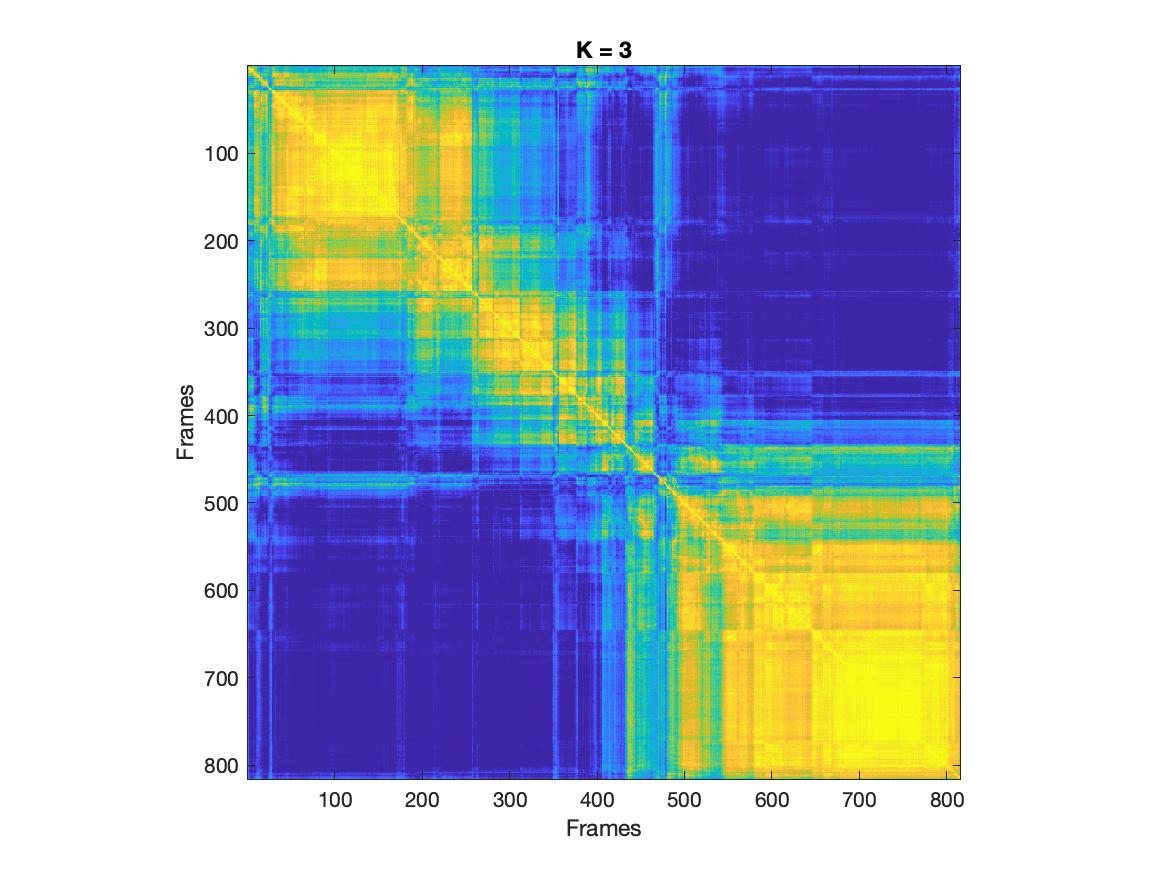

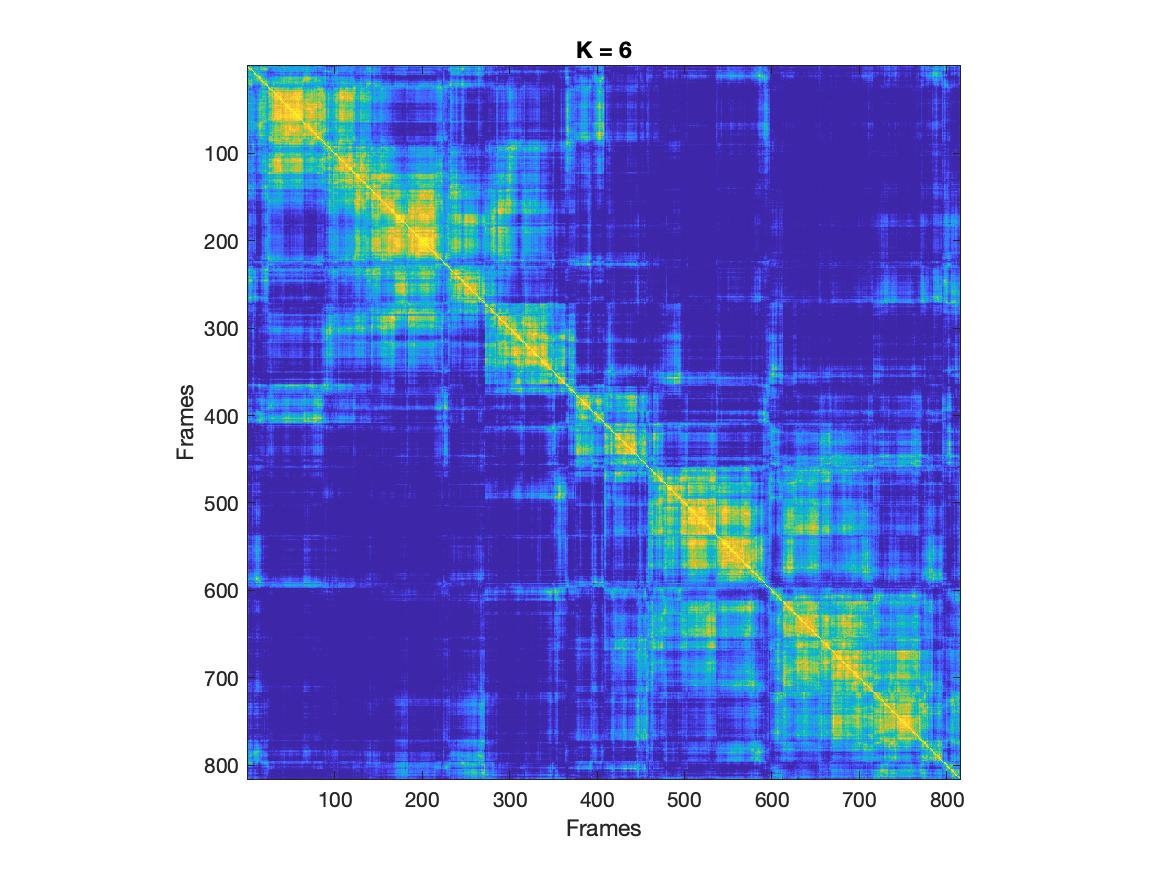

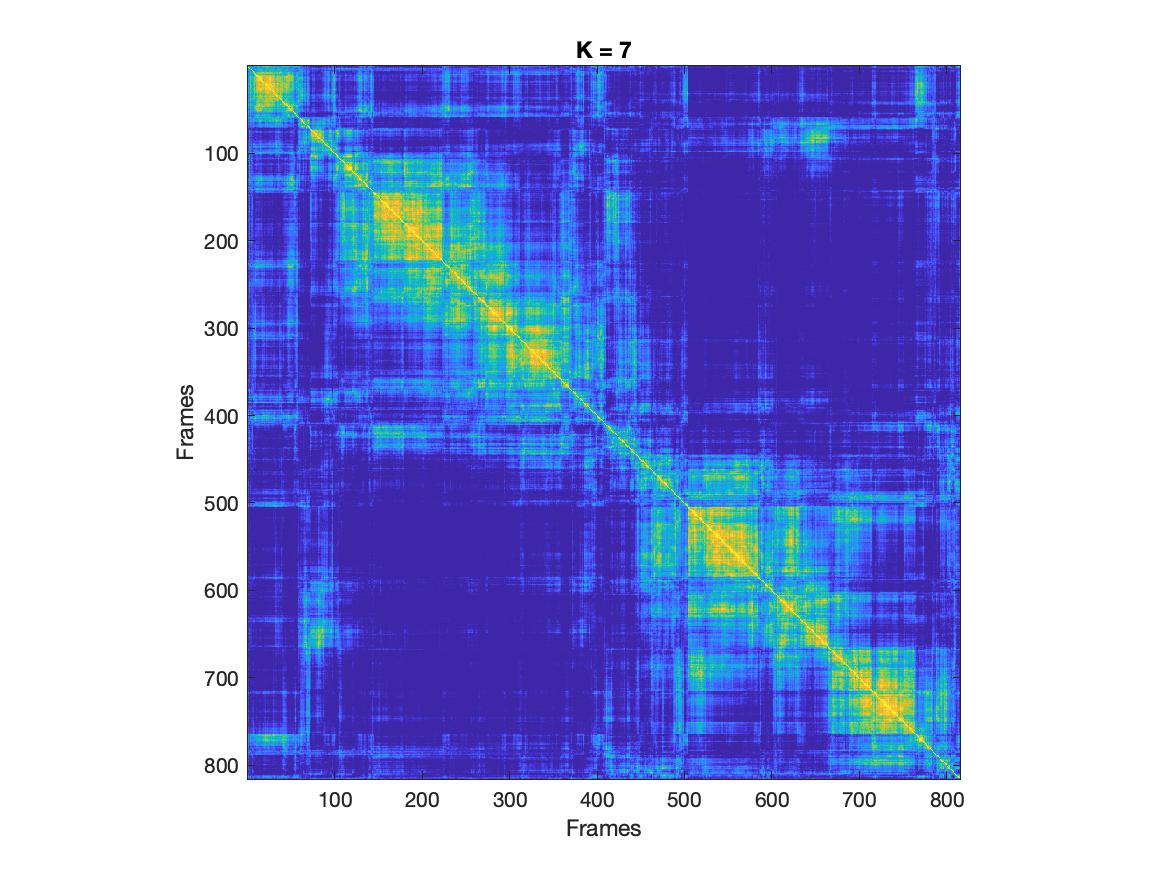

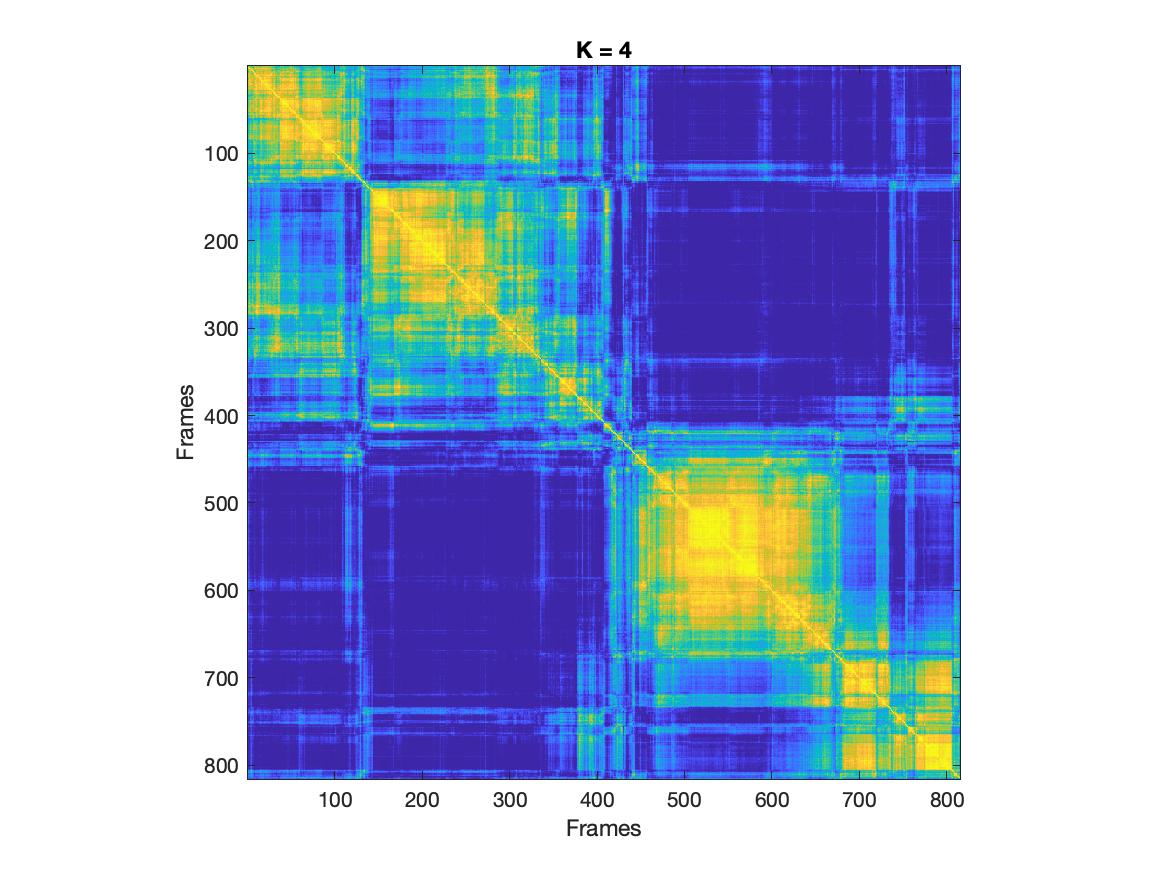


**Supplementary Figure 11. Co-activation pattern (CAP) maps based on nucleus ruber seed activation derived from healthy controls** (**A**) Three CAPs were selected. CAPs were z-scored and only the 15% most positive and 15% most negative contributions are represented in colour (z = ± 1.04), with red representing positive contributions and blue negative contributions. Locations are displayed in Montreal Neurological Institute (MNI) standard space coordinates. (**B**) Pie charts illustrating the percentage of positive and negative contributions within the 17 resting-state networks according to the convention of Yeo.^2^ Seed voxels were removed. Abbreviations: HC = healthy controls, Cont = Executive control, Default = Default mode, DorsAttn = Dorsal attention, Sal/VenAttn = Salience/Ventral attention, SomMot = somatomotor, TempPar = Temporoparietal, VisCen = Central vision, VisPer = Peripheral Visual.

**
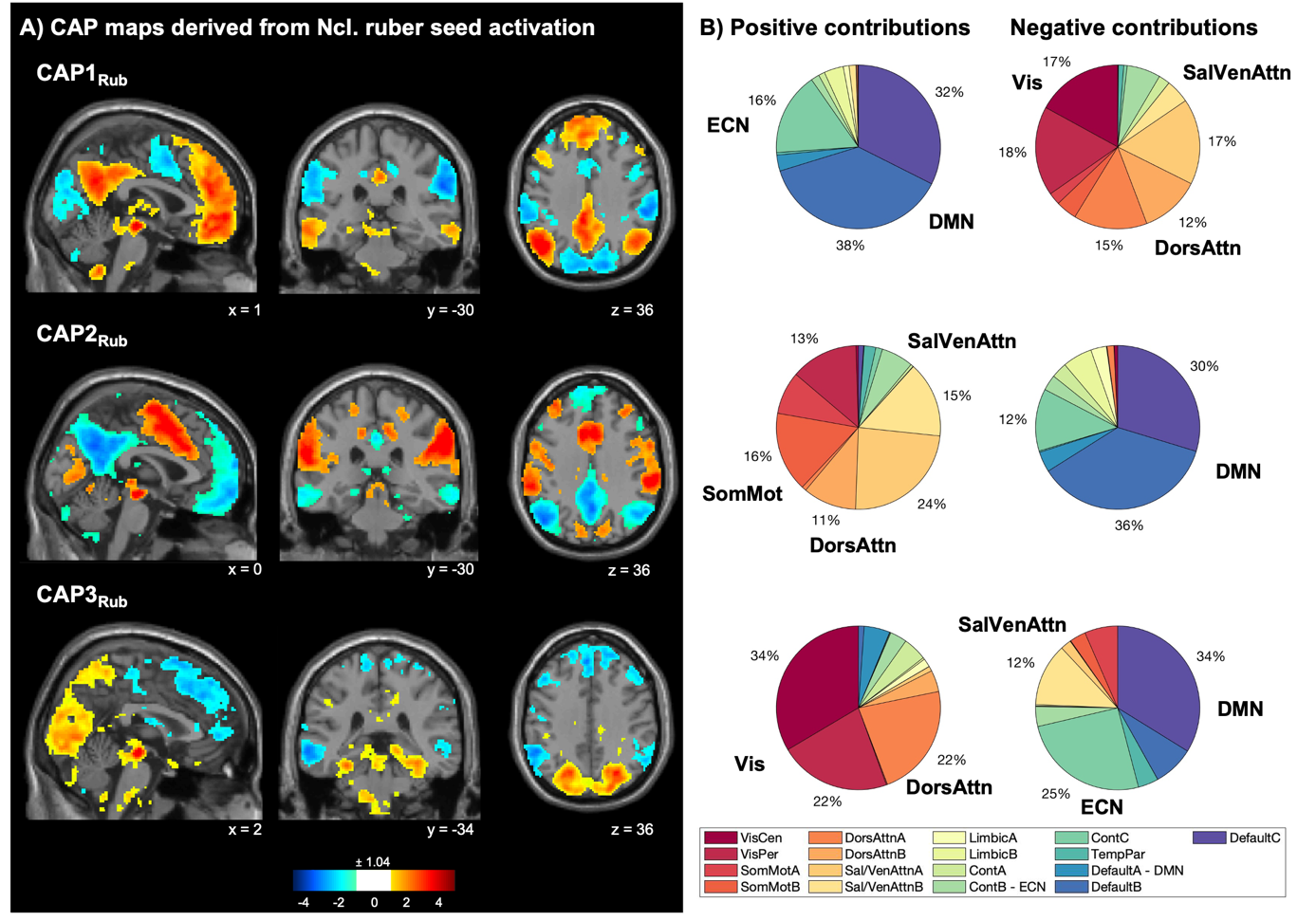
**

**Temporal Characteristics**

Adjusted for age, sex, number of discarded volumes, number of selected volumes, psychotropic medication, and depression and anxiety scores, group comparisons of temporal characteristics revealed that patients with dissociative seizures entered CAP1_Rub_ less often than dissociative seizure patients (*P* = 0.01). Lastly, patients did not significantly differ in their transition probabilities with respect to healthy controls.


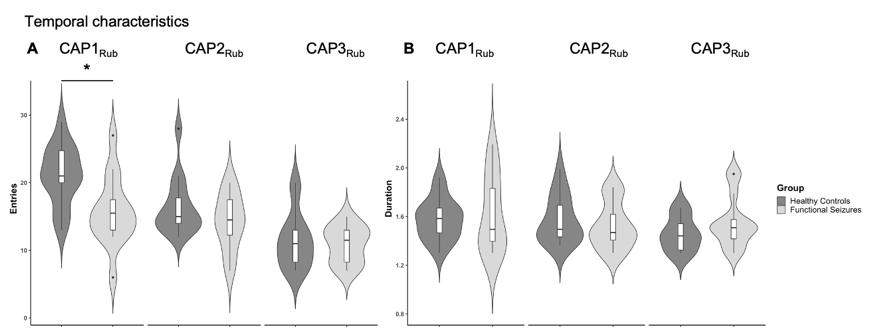


**Supplementary Figure 12. Co-activation pattern (CAP) temporal measures.** For CAPs derived from healthy controls compared to patients with dissociative seizures numbers of entries (right) and duration (left). Asterisks indicate statistical significance for data adjusted for covariates (i.e., number of excluded fMRI frames [derived from FD], number of selected frames [derived from CAPs], age, sex, psychotropic medication [dichotomous], depression [BDI] and anxiety [STAI-S] scores) and corrected for multiple comparisons. Boxplots: horizontal lines represent group median; box represents interquartile range and vertical line represents 1.5-times interquartile range. Violin plots visualize the distribution of the data. Abbreviations: BDI = Beck’s Depression Inventory, STAI = State-Trait Anxiety Inventory

**Supplementary Table 2. Statistical significance and effect sizes of transition probabilities between dissociative seizures patients and healthy controls.** The transition probability matrix contains *M* rows and *N* columns and shows the *P*-value and effect size (Cohen’s d) moving from state *M* to state *N*.

|  | CAP1_Rub_ | CAP2_Rub_ | CAP3_Rub_ |
| --- | --- | --- | --- |
| CAP1_Rub_ | 0.53 (0.27) | 0.60 (0) | 0.52 (0.39) |
| CAP2_Rub_ | 0.89 (0.63) | 0.61 (0.25) | 0.45 (0) |
| CAP3_Rub_ | 0.61 (0) | 0.62 (0) | 0.08 (-0.22) |

**References**

1. Șenbabaoğlu Y, Michailidis G, Li JZ. Critical limitations of consensus clustering in class discovery. *Sci Rep*. 2014;4(1):6207. doi:10.1038/srep06207

2. Yeo Th, Krienen FM, Sepulcre J, et al. The organization of the human cerebral cortex estimated by intrinsic functional connectivity. *J Neurophysiol*. 2011;106(3):1125-1165. doi:10.1152/jn.00338.2011
